# Transcriptomic and ultrastructural responses to Amiodarone–Itraconazole in naturally benznidazole-resistant and -susceptible *Trypanosoma cruzi* strains

**DOI:** 10.1371/journal.pntd.0013916

**Published:** 2026-01-14

**Authors:** Stivenn Gutiérrez, Carlos Ospina, Tatiana Cáceres, Luz Helena Patiño, Alberto Paniz-Mondolfi, Juan David Ramírez

**Affiliations:** 1 Centro de Investigaciones en Microbiología y Biotecnología-UR (CIMBIUR), School of Sciences and Engineering, Universidad del Rosario, Bogotá, Colombia; 2 Molecular Microbiology Laboratory, Department of Pathology, Molecular and Cell- based Medicine, Icahn School of Medicine at Mount Sinai, New York, New York, United States of America; 3 Center for Global Health and Interdisciplinary Research, USF Genomics Program, Department of Global, Environmental and Genomic Health Sciences, College of Public Health, University of South Florida, Tampa, Florida, United States of America; Universidade Federal de Sao Paulo, BRAZIL

## Abstract

Chagas disease (CD), caused by *Trypanosoma cruzi*, remains a major therapeutic challenge, primarily due to the limited efficacy of benznidazole and the emergence of naturally resistant strains. In this context, drug repurposing offers a promising strategy to identify compounds with trypanocidal activity. In this study, we evaluated the effect of Amiodarone-Itraconazole (Amiozole) against two *T. cruzi* strains belonging to DTU-TcI: one benznidazole-sensitive (MG) and one naturally resistant to benznidazol (DA). We employed an integrated approach combining transcriptomic and ultrastructural analyses to elucidate the compound’s mechanisms of action. Trypanocidal activity was assessed through cell viability assays (MTT), and IC_50_ values were determined using epimastigotes cultured in LIT medium. Subsequently, RNA sequencing was performed on treated samples, with reads mapped against the *T. cruzi* Dm28c reference genome. Differential gene expression was analyzed using DESeq2, followed by Gene Ontology enrichment analysis and metabolic pathway reconstruction via KAAS. In parallel, transmission electron microscopy (TEM) was used to evaluate ultrastructural alterations induced by treatment. Our results revealed susceptibility to Amiozole in both strains, although they exhibited markedly distinct transcriptomic responses. In the DA strain, 35 genes were upregulated and 87 downregulated, with notable activation of purine metabolism and inhibition of surface renewal pathways. In contrast, the MG strain showed 57 upregulated and 412 downregulated genes, including enhanced sphingolipid metabolism—potentially linked to membrane repair—and widespread suppression of energy and nucleotide biosynthesis pathways. At the subcellular level, both strains displayed severe damage, including mitochondrial disruption, nuclear disorganization, formation of autophagosomes, and extensive membrane vesiculation, reflecting multifocal cellular stress. Collectively, these findings provide a comprehensive view of Amiozole’s effects on *T. cruzi*, supporting a multifaceted mode of action that disrupts key biological processes essential for parasite viability. Our study underscores the potential of Amiozole as a combinatorial therapy against *T. cruzi* strains with distinct resistance profiles. Nevertheless, further research using infective forms, variable dosages, and diverse intra-DTU lineages is essential to validate its clinical applicability for Chagas disease.

## Author summary

Chagas disease is a serious parasitic infection that affects millions of people in Latin America and is spreading to other regions of the world. The current treatment, benznidazole, is not always effective and some parasite strains have developed natural resistance, which limits therapeutic options. To address this challenge, we tested a new drug mix called Amiozole, made of amiodarone and itraconazole, two drugs already approved for other uses. We studied how Amiozole affects two strains of the parasite *Trypanosoma cruzi*: one that is sensitive to benznidazole and another that is naturally resistant. Using a combination of RNA-sequencing analysis and high-resolution microscopy, we found that Amiozole damages the parasite in multiple ways, including its energy production, surface renewal, and structural integrity. Although both strains were susceptible, they responded in distinct ways at the molecular level, showing that the drug combination triggers complex stress responses. These results suggest that Amiozole could be a promising alternative treatment for CD. However, more studies are needed to confirm its safety and effectiveness in the infective stages of the parasite and in different parasite genetic populations.

## Introduction

*Trypanosoma cruzi*, the etiological agent of Chagas disease (CD), infects an estimated seven million people worldwide [1], with nearly 75 million at risk in Latin America [2]. Transmission occurs predominantly through hematophagous Triatominae vectors [3], although oral, congenital, laboratory exposure, transfusional, and transplant-associated routes are increasingly recognized, sustaining a complex epidemiological landscape. [4]. The parasite’s complex life cycle—comprising metacyclic trypomastigotes (MT), intracellular amastigotes (AM), cell-derived trypomastigotes (CDT), and epimastigotes (EP) [5]—underpins a disease that unfolds from an acute phase marked by high parasitemia to a chronic phase associated with progressive cardiomyopathy and megavisceral complications [6]. Despite its substantial public health burden, effective treatment options remain limited, and therapeutic failure linked to natural and acquired drug resistance continues to challenge disease control efforts.

Effective treatment options for CD remain severely limited, and therapeutic failure is increasingly linked to natural and acquired resistance across *T. cruzi* strains and discrete typing units (DTUs). Benznidazole (BNZ) and nifurtimox (NFX)—nitroheterocyclic compounds introduced more than five decades ago [7,8]—are still the only first-line therapies [1], yet both show variable efficacy, require prolonged regimens, and are associated with substantial toxicity [9,10]. Their antiparasitic activity relies on activation by parasite nitroreductases, which generate reactive intermediates that damage DNA, lipids, and proteins [11,12]. NFX induces oxidative stress through free-radical formation and thiol depletion, whereas BNZ forms electrophilic metabolites that alkylate macromolecules [13]. These mechanisms impose selective pressure that fosters drug resistance, while adverse effects—ranging from gastrointestinal and neurological toxicity with NFX to dermatological and sleep disturbances with BNZ [11]—further limit their clinical use. Research using BNZ as a biological probe has revealed detoxification, repair, and oxidative-stress pathways that enable parasite survival [14,15], including mechanisms present in naturally less susceptible strains such as MHOM/CO/01/DA [3]. Collectively, these deficiencies highlight the urgent need for safer, more effective, and resistance-resilient therapeutic alternatives.

In this context, repurposing or combining existing drugs has emerged as a promising strategy to enhance trypanocidal efficacy while mitigating toxicity. Amiodarone (AMD), a class III antiarrhythmic introduced in 1961 [16], exhibits a dual antiparasitic mechanism by disrupting *T. cruzi* calcium homeostasis and inhibiting ergosterol biosynthesis through oxidosqualene cyclase blockade [17]. Beyond its clinical benefit in managing arrhythmias in Chagas cardiomyopathy [18,19], AMD shows direct antiparasitic activity *in vitro* and in murine models with low host toxicity [20–22], and its combination with BNZ has demonstrated synergistic effects that modulate inflammation and preserve cardiac cell integrity [23,24]. Nevertheless, contrasting reports of absent efficacy or cytotoxicity in some systems [25] underscore the need to refine understanding of AMD’s mode of action. Itraconazole (ITZ), a C14α-sterol demethylase inhibitor, similarly targets sterol biosynthesis and disrupts multiple enzymatic pathways in parasite membranes [26]. Preclinical studies show strong activity *in vitro* and *in vivo*, with complete protection at low doses in mice [27], while clinical trials report a 53% parasitological cure rate and excellent long-term safety [28]. However, subsequent findings of strain-dependent susceptibility [29] and the demonstrated synergy of ITZ-BNZ combinations *in murine* and canine models [30,31] further emphasize the therapeutic potential—and the need for systematic characterization—of alternative or adjunctive treatments for CD.

Given the limited efficacy and growing resistance associated with current CD therapies, drug repositioning has emerged as a promising strategy to enhance trypanocidal activity while reducing treatment-related toxicity. The combination of amiodarone (AMD) and itraconazole (ITZ)—known as Amiozole and first proposed by Paniz-Mondolfi *et al*. (2009) [32]—has shown strong potential as an alternative or adjunctive therapy. *In vitro* studies demonstrate dose- and strain-dependent reductions in *T. cruzi* infection and replication with minimal toxicity to host cells [33], while canine models report improved clinical outcomes and increased survival [34,35]. Amiozole benefits from the complementary mechanisms of its components: AMD’s antiarrhythmic, anti-inflammatory, and sterol-biosynthesis-disrupting properties, together with ITZ’s inhibition of C14α-sterol demethylase, collectively induce profound biochemical alterations in *T. cruzi*. However, despite encouraging results, the molecular pathways and ultrastructural changes triggered by this combination remain poorly understood. Although transcriptomic studies have helped define drug susceptibility patterns [14,15], and ultrastructural analyses have identified damage signatures under individual drug pressure, no studies have yet integrated these approaches to evaluate the combined Amiozole response. Comprehensive molecular and structural characterization is therefore essential to elucidate the mechanisms underlying this promising therapeutic strategy.

Although characterizing alternative and repositioned drugs is essential to address the limited efficacy and emerging resistance associated with current CD treatments, elucidating their mechanisms of action and resistance requires approaches that extend beyond transcriptomics alone [36]. Gene expression profiling provides valuable information on parasite stress responses and susceptibility patterns, yet it cannot fully capture the downstream biochemical, structural, and organellar alterations that ultimately determine trypanocidal efficacy or resistance phenotypes [37]. Notably, previous transcriptomic studies have identified signatures and key stress-response genes in *T. cruzi* epimastigotes exposed to BNZ [15,38], but such analyses remain insufficient to explain functional and morphological consequences of drug pressure. Integrating transcriptomic data with ultrastructural examination therefore offers a more comprehensive framework to assess how the parasite responds to treatment at both the molecular and cellular levels [14,36,39,40]. This combined methodological strategy enhances detection of coordinated gene-expression changes, structural damage, and pathway-level perturbations that would remain undetected using a single approach, thereby strengthening the mechanistic foundation for evaluating AMD–ITZ combination therapy and supporting the development of more effective and resistance-resilient interventions for CD.

This study aims to deepen the understanding of how *T. cruzi* responds to Amiozole—a synergistic combination of amiodarone and itraconazole designed to overcome the limited efficacy and resistance associated with current therapies—by integrating transcriptomic and ultrastructural analyses. Because transcriptomics alone cannot fully resolve the mechanisms of action or resistance induced by antiparasitic drugs, we combined gene expression profiling with high-resolution examination of morphological and organellar alterations to capture both the molecular and cellular consequences of treatment. By evaluating strains with differing natural susceptibility to benznidazole, this approach enables the identification of coordinated structural damage and pathway-level transcriptional changes associated with pharmacological stress. We hypothesize that Amiozole induces a distinct, strain-dependent response in *T. cruzi*, reflected in both ultrastructural disruption and regulation of key biological processes, providing mechanistic insights that support the rational development of more effective and resistance-resilient therapeutic strategies for CD.

## Methods and materials

### Parasite culture

Two *T. cruzi* strains with differing BNZ susceptibility profiles were used: the MHOM/CO/04/MG strain, BNZ-sensitive, isolated from a chronic case in a 54-year-old woman from Yopal, Casanare (Colombia); and the MHOM/CO/01/DA strain, BNZ-resistant, obtained from an acute case in a pregnant woman from Miraflores, Boyacá (Colombia). Both strains were retrieved from the cryobank of the Microbiological Research Group at Universidad del Rosario (GIMUR) and cultured in 25 cm³ and 75 cm³ culture flasks using liver infusion tryptose (LIT) medium supplemented with 10% fetal bovine serum (FBS). Cultures were incubated at 26 °C and subcultured every 5 days to maintain parasites in the logarithmic growth phase, corresponding to the epimastigote (EP) stage.

### Determination of IC_50_ values for Amiodarone-Itraconazole treatments

For IC_50_ determination, Amiozole (Vida Pharmacal, Inc.), a fixed-dose commercial tablet containing itraconazole (50 mg) and amiodarone (150 mg) together with standard pharmaceutical excipients (e.g., microcrystalline cellulose, starch, and magnesium stearate), was used. Tablets were manually macerated under sterile conditions using a mortar and pestle to obtain a homogeneous powder, which was suspended in sterile 1 × phosphate-buffered saline (PBS), thoroughly vortexed, and homogenized to generate a 10 × stock solution. Working solutions were freshly prepared by serial dilution in LIT medium to yield final concentrations ranging from 50 to 0.19 µg/mL (192.13 μM to 0.73 μM). This concentration range was selected based on previous *in vitro* studies employing comparable exposure times (72 h) for IC_50_ determination in *T. cruzi* epimastigotes treated with benznidazole, as well as in related trypanosomatids such as *Leishmania infantum* and *L. major* exposed to benzthiazide, or albendazole under similar experimental conditions [38,41]. Importantly, the upper and intermediate concentrations also encompass drug levels that are biologically relevant to *in vivo* exposure, as reflected by veterinary studies in which dogs received amiodarone hydrochloride (~7.5 mg/kg, orally, once daily) with or without a loading dose and itraconazole (~10 mg/kg, orally, once daily), achieving steady-state plasma concentrations of approximately 1–2 µg/mL over long-term treatment [33,34]. Together, these considerations support the biological and translational relevance of the Amiozole dose range used in this study.

Once optimal EPs density was reached, parasites were quantified using a Neubauer chamber, and 1 × 10^6^ parasites/mL were seeded into 24-well plates. Cultures were exposed to the prepared Amiozole dilutions and incubated for 72 hours at 37 °C, a duration consistent with the MTT viability assay and representing a complete EP logarithmic growth cycle. Two controls were included in duplicate: (i) a positive growth control consisting of parasites in untreated LIT medium, and (ii) a negative control consisting of parasites exposed to hydrogen peroxide to induce cytotoxicity.

Cell viability was assessed using the colorimetric MTT assay (Abcam; protocol available at: https://www.abcam.com/en-us/technical-resources/protocols/mtt-assay), following the manufacturer’s instructions. Absorbance readings were obtained using a spectrophotometer, and the half-maximal inhibitory concentration (IC_50_) values were calculated by nonlinear regression using GraphPad Prism software v8.4.2.

#### Transcriptomic response evaluation.

**RNA extraction:** In a second experimental phase, *T. cruzi* cultures were treated at a concentration of 1 × 10^6^ parasites/mL in 25 cm³ culture flasks, using Amiozole at the previously determined IC_50_ concentration for each strain. Cultures were incubated at 26 °C for 72 hours. A total of 12 culture flasks were used, with three replicates per treatment and positive controls without drug exposure for both strains.

RNA extraction was performed from 12 total pellets using the RNeasy Plus Mini Kit (Qiagen, Düsseldorf, Germany), strictly following the manufacturer’s protocol. To assess RNA quality, 5 µL of eluate from each sample was taken: 2 µL were used for quantification and purity measurement by spectrophotometry (NanoDrop One/OneC, Thermo Scientific), and 3 µL were used to evaluate RNA integrity via 2% agarose gel electrophoresis. The remaining eluates were stored at -80 °C until further processing.

**Library preparation and RNA sequencing:** Total RNA samples that passed quality control, as assessed by 2% agarose gel electrophoresis and Qubit fluorometric quantification (Thermo Fisher Scientific), were submitted to Novogene Bioinformatics Technology Co., Ltd. (Sacramento, USA) for library preparation and sequencing. Libraries were constructed using the TruSeq Stranded mRNA kit (Illumina), incorporating poly(A) mRNA enrichment via oligo(dT) selection and rRNA depletion. Sequencing was performed on an Illumina HiSeq X-TEN platform in paired-end mode (2 × 150 bp), generating a minimum sequencing depth of 9 Gb per sample. Raw read quality was evaluated using FastQC v0.11.9, followed by adapter trimming and quality filtering to remove reads containing more than 10% ambiguous bases (N) and reads with a Phred quality score below Q20. All raw sequencing reads generated in this study have been deposited in the European Nucleotide Archive (ENA) under the project accession PRJEB97688. Filtered reads were stored on the institutional server at Universidad del Rosario for subsequent bioinformatic analyses.

**Bioinformatic preprocessing:** Transcriptomic analysis was conducted using the data generated by Novogene following a standard workflow. Initially, raw read quality was assessed using FastQC and MultiQC (https://www.bioinformatics.babraham.ac.uk/projects/fastqc/). Due to detected adapter contamination, reads were processed with Trimmomatic (http://www.usadellab.org/cms/?page=trimmomatic), applying the parameters SLIDINGWINDOW:4:20, MINLEN:100, and AVGQUAL:20.

Filtered reads were aligned to the reference genome *Trypanosoma cruzi* Dm28c available in the NCBI genome database (https://www.ncbi.nlm.nih.gov/datasets/genome/GCA_003177105.1/) using STAR v2.7.10b (https://github.com/alexdobin/STAR) with two-pass alignment mode enabled *(--twopassMode Basic*) and restricting multiple alignments *(--outFilterMultimapNmax* 1).

Gene-level quantification was performed using featureCounts (Subread v2.0.3) with the corresponding GTF annotation file. The tool was run in paired-end mode (-*p*), requiring properly paired fragments (-*B*), and consistent orientation (-*C*), generating a gene count matrix from the sorted BAM files.

**Differential expression analysis:** Differential gene expression analysis between the DA and MG strains was performed using the DESeq2 package (https://github.com/thelovelab/DESeq2) within the R statistical environment (version 2024.12.0). Initially, the count matrix was loaded and experimental conditions were defined to construct a DESeqDataSet object. Statistical analysis was conducted using the DESeq method, which employs a generalized linear model with a negative binomial distribution to normalize read counts and assess the statistical significance of differentially expressed genes (DEGs) between treated and untreated conditions. DEGs were identified based on criteria of |log2FoldChange| ≥ 1.5 and an adjusted p-value (Benjamini-Hochberg) < 0.05.

For result visualization and validation, four graphical representations were generated: (i) Principal Component Analysis (PCA) plots constructed with ggplot2 (v3.5.1; https://ggplot2.tidyverse.org); (ii) Volcano plots created using the enhancedVolcano package in R (v1.24.0; https://github.com/kevinblighe/EnhancedVolcano); (iii) Venn diagrams generated with the VennDiagram package (v1.7.3; https://github.com/uclahs-cds/package-VennDiagram); and (iv) Heatmaps produced using the pheatmap package (v1.0.12; https://github.com/raivokolde/pheatmap).

**Gene ontology and biochemical pathway reconstruction:** Functional enrichment analysis was performed using the Gene Ontology (GO) annotations available in TriTrypDB (https://tritrypdb.org/tritrypdb/app) to identify biological processes (BP), cellular components (CC), and molecular functions (MF) associated with differentially expressed genes (DEGs), considering an adjusted p-value < 0.05. Results were visualized using bubble plots generated with the ggplot2 package in R. For biochemical pathway reconstruction in *T. cruzi*, upregulated and downregulated genes in the DA and MG strains were analyzed using the KAAS (KEGG Automatic Annotation Server; https://www.genome.jp/kaas-bin/kaas_main) online tool. The pathway data were subsequently represented with palette plots created using ggplot2 in R.

#### Electron microscopy.

In a third experimental stage, EPs were treated with Amiozole at a concentration corresponding to the previously determined IC_50_, using a parasite density of 1 × 10^6^ parasites/mL and incubated for 72 hours. After treatment, samples were fixed with glutaraldehyde to preserve cellular ultrastructure and subjected to a progressive dehydration process through a graded ethanol series. Subsequently, samples were embedded in epoxy resin for polymerization. Ultrathin sections were obtained using an ultramicrotome equipped with a diamond knife and mounted on copper grids. Contrast staining was performed with uranyl acetate and lead citrate solutions. Finally, the samples were examined by transmission electron microscopy (TEM) using a Hitachi H7800 microscope at the Icahn School of Medicine at Mount Sinai, New York, USA.

## Results

### Susceptibility to treatment and quality of transcriptomic data

The half-maximal inhibitory concentration (IC_50_) values for Amiozole were 3.64 µg/mL for the naturally BNZ-resistant DA strain and 2.32 µg/mL for the BNZ-susceptible MG strain (S1 Fig), indicating comparable sensitivity to this drug combination. Although the IC_50_ values differed modestly between strains, this variation was limited and fell within a similar low-micromolar range, supporting classification of both strains as susceptible under the experimental conditions tested. Importantly, when contrasted with the markedly divergent IC_50_ values obtained for the same strains exposed to BNZ—28.92 µg/mL (111.13 µM) for DA and 0.88 µg/mL (3.39 µM) for MG [38]—the Amiozole response showed a substantially narrower susceptibility gap (S1 Fig). This relative convergence in inhibitory concentrations suggests that Amiozole may partially overcome strain-dependent differences observed with first-line therapy, supporting the use of these strains as a relevant comparative model for evaluating alternative or combination treatments.

Furthermore, RNA sequencing of these strains following 72 hours of Amiozole exposure yielded approximately 12 million reads per sample across the 12 samples analyzed in this study (6 samples corresponding to Amiozole-treated DA and MG strains, and 6 untreated parasite samples for both strains serving as negative controls).

### Gene expression changes induced by Amiozole

At 72 hours post-treatment with Amiozole, distinct patterns of differential gene expression were observed between the DA and MG strains. For each strain, untreated parasites served as controls and references for identifying differentially expressed genes (DEGs).

**Fig 1:**
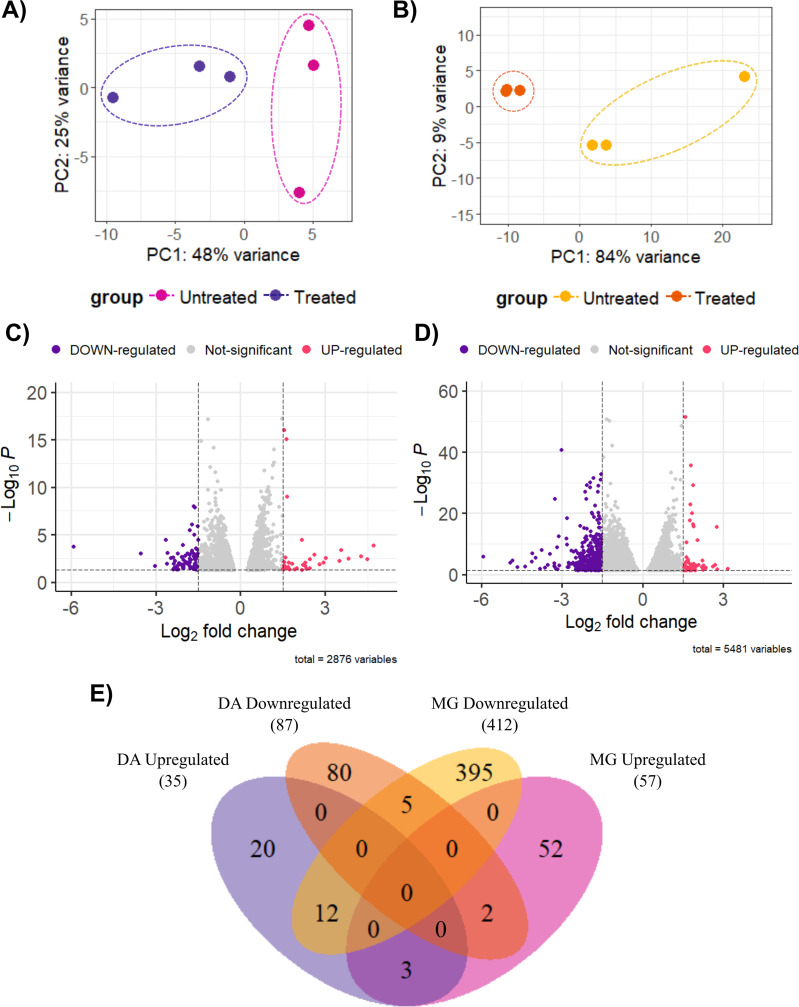
Differential gene expression analysis in DA and MG strains after 72 hours of Amiozole exposure. Principal component analysis (PCA) for DA **(A)** and MG **(B)** strains comparing treated and untreated conditions. Volcano plots display differentially expressed genes (DEGs) between treated and untreated parasites in DA **(C)** and MG **(D)**; magenta dots represent upregulated genes, purple dots indicate downregulated genes, and gray dots correspond to genes with no significant change. **(E)** Venn diagram summarizing the total number of DEGs in both strains, distinguishing upregulated, downregulated, and shared genes across categories.

As an initial approach, principal component analysis (PCA) was performed to assess the overall variation in gene expression between treated samples and controls. In the DA strain, the PCA revealed a clear separation between treated and untreated groups, although two replicates per group showed some dispersion, which reduced the variance explained by the first two principal components (PC1: 48%; PC2: 25%) (Fig 1A). In contrast, the MG strain displayed a more compact clustering of treated samples, while the untreated samples showed a dispersion pattern similar to that observed in DA, with a higher proportion of variance captured by PC1 (84%) and PC2 (9%) (Fig 1B). These results indicate a consistent transcriptomic response induced by Amiozole treatment, particularly pronounced in the drug-sensitive strain.

The differential expression analysis identified a total of 13,513 differentially expressed genes (DEGs) in the DA strain and 13,622 in the MG strain (adjusted p-value < 0.05). Within these, 678 genes were upregulated and 527 downregulated in DA, whereas MG exhibited 2,036 upregulated and 2,400 downregulated genes.

Applying more stringent criteria (log_2_FoldChange| ≥ 1.5 and adjusted p-value < 0.05), 122 DEGs were identified in DA (35 upregulated and 87 downregulated) (Fig 1C), and 469 DEGs in MG (57 upregulated and 412 downregulated) (Fig 1D). This pattern indicates a stronger transcriptomic repression in both strains, particularly pronounced in MG. Finally, a subset of DEGs was shared between the two strains, potentially representing a conserved transcriptional response to Amiozole treatment (Fig 1E).

### Functional annotation of genes

**Fig 2:**
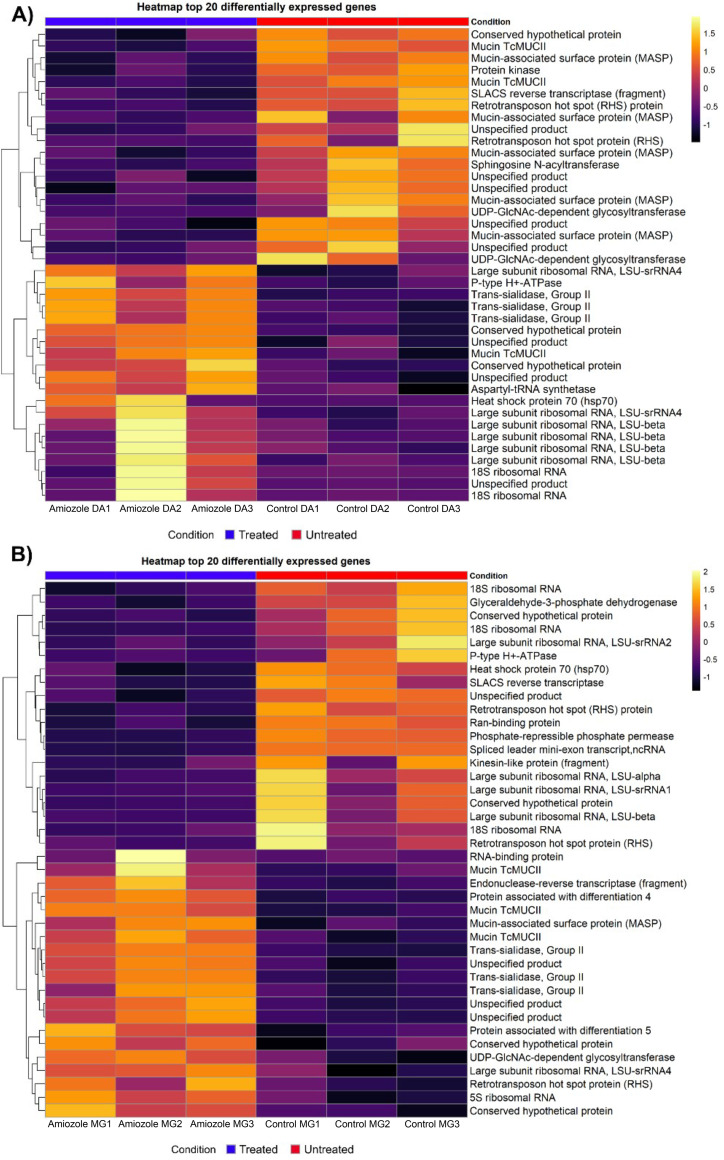
Heatmaps of the top 20 upregulated and downregulated genes (Log2FoldChange) in DA and MG strains treated with Amiozole. The heatmaps display expression levels of the 20 genes with the greatest differential expression in the DA **(A)** and MG **(B)** strains. Colors indicate gene expression changes (Log2FoldChange) following a gradient from yellow to purple, representing increased to decreased expression, respectively. Rows correspond to individual genes, and columns represent different biological replicates.

Functional annotation of differentially expressed genes (DEGs) in the DA and MG strains was performed using TriTrypDB, and transcriptomic patterns were summarized using heatmaps displaying the 20 genes with the highest absolute log₂FoldChange values. In the DA strain, Amiozole-treated samples were characterized by increased abundance of ribosomal transcripts, including multiple large subunit rRNAs (LSU-β, LSU-α, and LSU-srRNAs) and 18S rRNA, indicating a strong modulation of the translational machinery (S1 Table). In contrast, untreated DA samples showed relatively higher expression of RHS (retrotransposon hot spot) proteins and UDP-GlcNAc pyrophosphorylase, which were therefore highlighted as representative non-ribosomal features within this subset (Fig 2A). In the MG strain, the Amiozole-treated sample displayed prominent upregulation of surface-associated gene families, particularly TcMUCII mucins, MASPs, trans-sialidases, and GP63 proteases, alongside a limited number of regulatory transcripts such as a single RNA-binding protein and an endonuclease–reverse transcriptase fragment (S2 Table). Ribosomal RNAs and RHS proteins were more abundant in untreated MG controls (Fig 2B). Overall, these results indicate that the most extreme transcriptional responses are dominated by ribosomal and surface protein gene families, and the genes emphasized in the text reflect representative functional categories within this constrained gene set rather than an exhaustive mechanistic interpretation.

Regarding the most repressed genes in the DA strain, the sample ‘Amiozole DA1’ exhibited marked downregulation of genes encoding ribosomal components such as LSU-beta and 18S ribosomal RNA, as well as genes related to MASP and nonspecific products (S1 Table). In the ‘Amiozole DA3’ sample, decreased expression was observed for genes encoding heat shock proteins, including HSP70. Conversely, control samples showed elevated expression of conserved and nonspecific genes, including MASP proteins, RHS, and UDP-GlcNAc pyrophosphorylase (Fig 2A). In the MG strain, the treated sample ‘Amiozole MG2’ displayed differential regulation of genes coding for HSP70, GAPDH, RHS proteins, and other nonspecific products (S2 Table). Untreated controls tended to exhibit downregulation of genes such as LSU-srRNA4, UDP-GlcNAc pyrophosphorylase, MASP, trans-sialidases, and conserved hypothetical proteins (Fig 2B). Together, these findings reflect a differential modulation of key genes associated with parasite metabolism, ribosomal machinery, and surface molecules.

### Functional Enrichment Analysis (GO)

**Fig 3:**
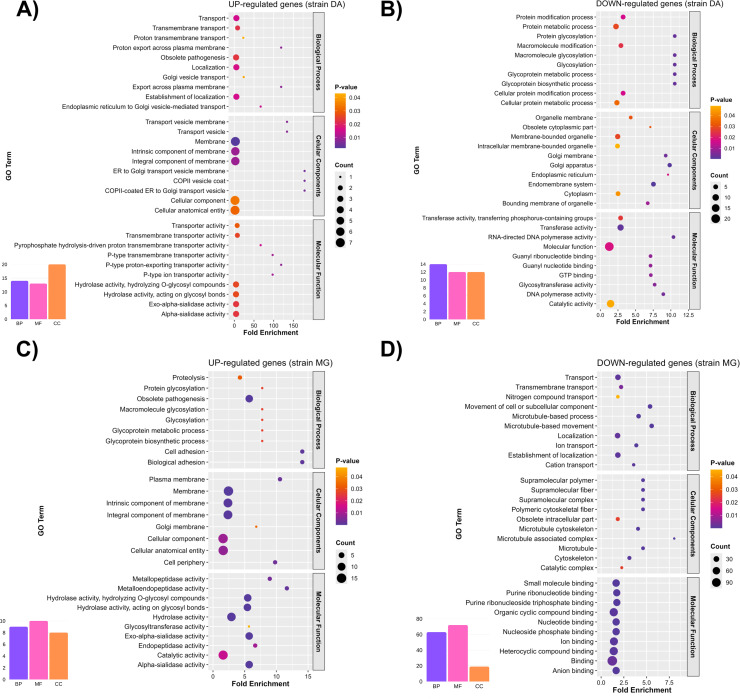
Enrichment of Gene Ontology (GO) terms in differentially expressed genes from DA and MG strains. A functional enrichment analysis based on GO was conducted to identify biological processes (BP), cellular components (CC), and molecular functions (MF) significantly associated with differentially expressed genes (DEGs) in the DA and MG strains following Amiozole treatment. Each plot displays the most representative GO terms based on the number of annotated genes (point size), enrichment score (X-axis), and statistical significance (color intensity, adjusted p-value). Panels are organized as follows: **(A)** upregulated genes in DA; **(B)** downregulated genes in DA; **(C)** upregulated genes in MG; and **(D)** downregulated genes in MG. Venn diagrams summarizing the total number of GO terms identified by functional category are included in each panel.

Gene Ontology (GO) term enrichment analysis revealed marked differences between the DA and MG strains. In DA, 47 terms were upregulated and 38 downregulated, whereas in MG, 27 terms were upregulated and 154 downregulated (adjusted p-value < 0.05), indicating a broader functional repression in the sensitive strain (Fig 3A–3D). The enrichment plot highlighted the top 30 terms based on gene count, enrichment, and statistical significance. In the DA strain, upregulated genes were associated with biological processes such as ER-Golgi vesicular transport and export of molecules across the plasma membrane, while downregulated genes were mainly linked to biosynthesis and post-translational modifications like protein glycosylation. Within the cellular component category, upregulated DEGs were related to the plasma membrane and the ER-Golgi complex, contrasting with downregulated genes affecting intracellular structures and organelles. The most enriched molecular functions included transmembrane transport activities and hydrolase functions, whereas repressed genes were associated with replication, genomic maintenance, surface protein modification, and cell signaling.

In the MG strain, upregulated DEGs showed significant enrichment in parasite-host interaction processes and protein glycosylation, while downregulated genes were associated with essential functions of intracellular transport, ion trafficking, and microtubule-based motility. Regarding cellular components, upregulated genes were mainly linked to plasma membrane structures and the Golgi apparatus, whereas downregulated genes exhibited a clear association with the cytoskeleton, including microtubules and supramolecular fibers. In terms of molecular functions, there was a notable enrichment of hydrolase and peptidase activities involved in the modification of glycoconjugates such as mucins and glycoproteins—key factors for immune evasion and oxidative stress response—while repressed genes were related to binding functions for small ligands such as nucleotides, ions, and heterocyclic compounds, which are fundamental for signaling, energy metabolism, and DNA replication (Fig 3C and 3D).

### Metabolic pathway analysis

The enrichment analysis of metabolic pathways revealed distinct transcriptomic profiles between the BNZl-resistant DA strain and the sensitive MG strain following treatment. In the DA strain, only one pathway was significantly upregulated: purine metabolism (fold enrichment = 3.89; p = 0.0312), suggesting a possible adaptive response to the drug. However, marked repression was observed across multiple metabolic pathways, primarily related to carbohydrate and complex lipid metabolism, including sphingolipid metabolism (fold enrichment = 2.54; p = 0.0103), fructose and mannose metabolism (fold enrichment = 5.95; p = 0.0026), and several pathways associated with glyconjugate biosynthesis (glycosphingolipids, O-type mucins, and GPI anchors), with fold enrichment values exceeding 8 and highly significant p-values (p < 0.005), indicating strong suppression of these biosynthetic functions.

Conversely, in the MG strain, an opposite pattern was observed, with sphingolipid metabolism being significantly upregulated (fold enrichment = 3.39; p = 1.42E-06), contrasting with its repression in DA and suggesting a differential mechanism of adaptation to the treatment. Concurrently, multiple downregulated metabolic pathways related to energy and nucleotide metabolism were identified, including nitrogen metabolism (fold enrichment = 10.81; p = 3.64E-07), taurine and hypotaurine metabolism (fold enrichment = 11.8; p = 6.82E-06), and key pathways for sugar metabolism (starch and sucrose), as well as nucleotide, folate, riboflavin, and thiamine biosynthesis. This coordinated repression reflects a metabolically compromised state induced by the treatment in the sensitive strain.

**Fig 4:**
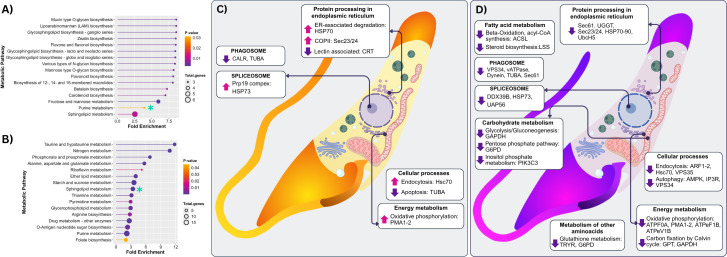
Metabolic pathway analysis in DA and MG strains following Amiozole treatment. **(A)** Palette plot depicting upregulated and downregulated metabolic pathways in the DA strain, indicating the total number of genes involved, fold enrichment, and adjusted p-value for each pathway. **(B)** Equivalent plot for the MG strain using the same parameters and analysis criteria. **(C–D)** Schematic representation of the main altered metabolic pathways derived from the functional analysis of DEGs, illustrating their integration into the biology of Amiozole-treated parasites in DA **(C)** and MG **(D)**.

Finally, the graphical summary in (Fig 4C and 4D) highlights the transcriptomic impact of Amiozole on key metabolic and cellular pathways in both strains. Upregulation of genes involved in protein processing within the endoplasmic reticulum (HSP70, Sec23/24), the spliceosome (HSP73), endocytosis (Hsc70), and oxidative phosphorylation (PMA1–2) was observed (Fig 4C). In contrast, a strong common repression was identified in essential pathways related to carbohydrate metabolism (GAPDH, G6PD, PIK3C3), energy production (ATP synthases, Calvin cycle), and fatty acid metabolism (ACSL), along with disruptions in phagosomes, autophagy, and the glutathione antioxidant pathway (Fig 4D). These findings suggest a complex response combining focal activation of critical functions with a widespread suppression of biosynthetic and energetic processes vital for parasite survival.

### Ultrastructural alterations observed by transmission electron

**Fig 5:**
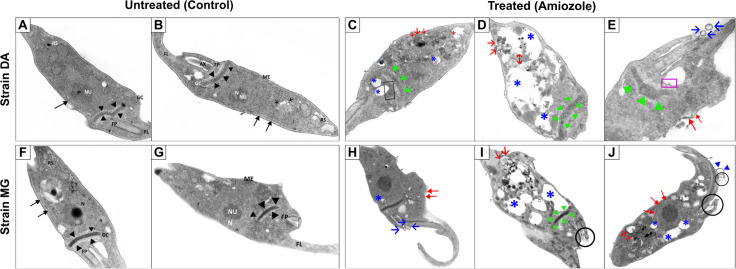
Ultrastructural alterations induced by Amiozole in DA (resistant) and MG (sensitive) strains of *T. cruzi*, observed by transmission electron microscopy (TEM) after 72 h of treatment. **(A, B)** Untreated DA parasites exhibit preserved cellular architecture, showing a compact kinetoplast positioned between the nucleus and the flagellar pocket, well-defined reservosomes, and an adherent flagellum. **(C–E)** Amiozole-treated DA parasites display significant ultrastructural damage, including abundant autophagosome formation (blue asterisk), detachment of reservosomes (open red arrow), Golgi complex disruption (black box), loss of kinetoplast compaction (green triangle), cytoplasmic autophagic vacuoles (red cross), membrane vesiculation (double-headed red arrow), and abnormal plasma membrane structures (red arrow). **(F, G)** Untreated MG parasites show preserved morphological integrity, with a compact kinetoplast, defined nucleolus, and organized Golgi complex. **(H–J)** Treated MG parasites exhibit severe alterations such as mitochondrial swelling and loss of perinuclear integrity (red arrow), autophagosome formation, reservosome detachment, kinetoplast disorganization, plasma membrane vesicle formation (open blue arrow), vesicles within the flagellar pocket (black circle), and abnormal membrane projections (blue triangle). RS, reservosomes; NU, nucleolus; Black arrow, mitochondrion; Black triangle, kinetoplast; FL, flagellum; AX, axoneme; FP, flagellar pocket; ME, membrane; GC, Golgi complex; N, nucleus; Open arrow, lipid inclusions.

Transmission electron microscopy (TEM) analysis of the resistant DA and sensitive MG strains of *T. cruzi* after 72 hours of exposure to Amiozole treatment revealed marked alterations in their ultrastructural architecture compared to their respective untreated controls. In untreated DA parasites (Fig 5A and 5B), preserved cellular organization was observed, characteristic of the epimastigote stage, including a compact kinetoplast located between the nucleus and the flagellar pocket, well-defined reservosomes, and a flagellum attached to the posterior region of the cell body. However, DA parasites treated with Amiozole exhibited multiple ultrastructural damages, including abundant formation of autophagosomes, detachment of reservosomes, disruption of the Golgi apparatus, and loss of kinetoplast compaction (Fig 5C). Additionally, anomalous structures in the plasma membrane (Fig 5D), cytoplasmic autophagic vacuoles, intravesicular vesicles, and membrane vesiculation were observed (Fig 5E).

Similarly, in the untreated MG strain, characteristic intracellular structures were preserved, including a compact kinetoplast, a clearly defined nucleolus within the nucleus, and a Golgi complex composed of multiple stacked membranes (Fig 5F and 5G). However, following treatment with Amiozole, severe alterations were identified, including swelling and loss of integrity of the perinuclear space, as well as vesicle formation in the plasma membrane (Fig 5H). Additionally, autophagosomes, detachment of reservosomes, and disorganization of the kinetoplast were observed (Fig 5I), along with vesicle formation in the flagellar pocket and anomalous membrane projections (Fig 5J). These observations suggest that Amiozole treatment induces profound cellular damage, affecting structures essential for parasite viability and morphological organization in both Benznidazole-sensitive and -resistant strains.

Amiozole treatment elicited strain-specific responses in *T. cruzi*, with distinct transcriptomic and ultrastructural profiles between resistant (DA) and sensitive (MG) strains. The DA strain activated compensatory pathways involving vesicular trafficking and purine metabolism while suppressing biosynthetic functions, suggesting targeted adaptation. In contrast, MG showed broad downregulation of critical processes including intracellular transport, energy metabolism, and structural maintenance. Ultrastructural analysis corroborated these differences: DA exhibited autophagy-related modifications consistent with adaptive responses, while MG displayed severe cellular disorganization reflecting systemic damage. These findings demonstrate that benznidazole resistance does not confer cross-resistance to Amiozole, with DA maintaining coordinated transcriptional regulation compared to MG’s global dysregulation.

## Discussion

CD remains a critical public health challenge in Latin America, largely due to the limited efficacy of current treatment options—exclusively based on benznidazole and nifurtimox—whose use is constrained by toxicity, prolonged duration, and low effectiveness during the chronic phase [6,42]. These limitations are compounded by the emergence of drug-resistant *T. cruzi* strains, which are associated with treatment failure and poor adherence, further reducing therapeutic options [37]. Given the chronic underfunding and lack of investment in new therapies or vaccines—a hallmark of neglected tropical diseases—drug repurposing has emerged as a feasible and urgently needed strategy [22]. In this study, we evaluated the effect of a combinatorial treatment using amiodarone and itraconazole on *T. cruzi* strains with contrasting benznidazole sensitivity profiles (DA: resistant; MG: sensitive), integrating high-throughput transcriptomics (RNA-seq) and transmission electron microscopy to characterize the molecular and ultrastructural responses induced by the treatment.

Differential gene expression analysis at 72 hours post-treatment with Amiozole revealed significant transcriptomic modulation in both *T. cruzi* strains. Principal component analysis (PCA) showed a clear separation between treated and control groups, indicating a global impact of the drug on gene expression. However, in the DA strain, some samples exhibited lower coherence across principal components, possibly reflecting the intrinsic genetic variability of *T. cruzi*—a known limitation previously reported in studies involving wild-type populations and strains with natural or induced resistance to BNZ, as well as in related models such as *Leishmania* [14,43,44]. Importantly, the genetic basis of BNZ resistance in this strain has been partially characterized in a recent transcriptomic study by Ospina et al. (2025), where an IC₅₀ of 28.92 µg/mL was associated with enrichment of genes involved in amino acid metabolism, DNA repair, redox homeostasis, and protein translation, suggesting survival strategies that extend beyond generalized stress responses [38]. In this study, all transcriptomic analyses were performed at a single Amiozole concentration corresponding to the IC_50_; therefore, the variability observed among replicates cannot be ascribed to dose-dependent effects. Instead, this heterogeneity likely reflects intrinsic biological factors, including population asynchrony, differential stress adaptation, and the strong post-transcriptional regulatory landscape characteristic of *T. cruzi*. At the IC_50_ level, parasites may occupy distinct physiological states in which some cells activate compensatory or stress-related programs, whereas others undergo more pronounced functional impairment. Such variability has been reported previously for trypanocidal drugs and underscores that transcriptomic responses at a fixed inhibitory concentration capture a spectrum of adaptive states rather than a uniform cytotoxic outcome [45].

Differential gene expression analysis at 72 h post-treatment with Amiozole revealed substantial transcriptomic modulation in both *T. cruzi* strains, with PCA clearly separating treated and control groups, consistent with a global drug-induced response. Nonetheless, the DA strain displayed greater dispersion among treated replicates, which is more plausibly explained by intrinsic biological and genetic heterogeneity than by experimental variation, given that all samples were exposed to a single IC_50_ concentration. Such variability has been widely reported in *T. cruzi* and related kinetoplastids and is particularly evident in strains with natural or acquired drug resistance, where diverse adaptive and post-transcriptional regulatory programs may be differentially engaged [14,43,44]. In this context, prior transcriptomic analyses of the DA strain have shown that BNZ resistance is associated with heterogeneous activation of pathways linked to metabolism, DNA repair, redox balance, and translation, underscoring the existence of multiple survival strategies within the same strain [38]. Our results therefore suggest that the differences observed between replicates reflect strain-specific plasticity in stress adaptation under Amiozole pressure rather than dose-dependent effects, highlighting the complexity of drug responses in genetically diverse *T. cruzi* populations.

Applying stringent thresholds for biological significance (Log_2_FC ≥ 1.5 or ≤ –1.5; *p* < 0.05) markedly reduced the number of differentially expressed transcripts, revealing a dominant trend toward gene downregulation in both strains. Rather than implying direct inhibition of transcription, this global repression is more appropriately interpreted as a stress-associated remodeling of mRNA abundance within the context of the strong post-transcriptional regulatory landscape that characterizes *T. cruzi*. Alternatively, it may highlight the limited trypanocidal potency of amiodarone as monotherapy, as previously reported [25,46]. Notably, the susceptible MG strain exhibited a higher proportion of repressed genes, potentially indicative of an acute compensatory shutdown aimed at safeguarding core functions—an effect also described in BNZ-resistant strains [15]. The identification of commonly regulated transcripts across both strains points to the activation of partially conserved stress-response circuits, supporting the notion that BNZ resistance does not confer cross-tolerance to Amiozole but instead elicits divergent adaptive strategies. This raises a critical question: do these transcriptomic signatures reflect strain-specific modulation of cellular programs under Amiozole pressure? Addressing this will require integrative multi-omic analyses—including transcriptomic, proteomic, and metabolomic profiling—to unravel the downstream functional consequences of treatment [47,48]. In parallel, genome-wide association studies (GWAS) across diverse clinical isolates could reveal genetic variants linked to Amiozole sensitivity or tolerance [49], while quantitative pharmacodynamic studies will be essential to define therapeutic thresholds, discover predictive biomarkers, and guide the design of optimized drug combinations [37].

Functional annotation of differentially expressed genes in *T. cruzi* strains DA and MG revealed distinct molecular adaptations to Amiozole exposure. In strain DA, the upregulation of ribosomal components such as LSU-β and 18S rRNA suggests an effort to sustain protein synthesis under stress conditions [50]. In contrast, untreated controls predominantly expressed transcripts encoding RHS proteins and UDP-GlcNAc pyrophosphorylase—molecules implicated in genome remodeling and surface glycoprotein modification [51]. These genes, belonging to expansive multigene families located in telomeric and subtelomeric regions, exhibit strain-specific expression patterns that likely shape *T. cruzi*’s genomic plasticity and transcriptional flexibility [52,53]. Conversely, strain MG displayed a more heterogeneous transcriptomic response, marked by the upregulation of RNA-binding proteins and TcMUCII-type mucins, possibly involved in post-transcriptional regulation and surface reinforcement under stress [54,55]. Mucins, also part of large multigene families, are closely linked to virulence, pathogenicity, and metacyclogenesis, and are encoded in dynamic chromosomal regions that favor gene regulation and parasite persistence [53]. Notably, untreated MG controls exhibited elevated ribosomal activity, indicative of a highly active basal metabolism. Together, these contrasting profiles highlight the parasite’s adaptive plasticity and suggest that the response to Amiozole involves a dynamic interplay between maintaining translational capacity, activating stress responses, and remodeling the cell surface. Future functional studies targeting these genes may clarify their roles in drug tolerance and support the identification of therapeutic targets that account for *T. cruzi*’s intraspecific diversity.

Amiozole-induced gene repression reveals critical disruptions in *T. cruzi* homeostasis; however, these changes should be interpreted within the context of the parasite’s predominantly post-transcriptional gene regulation. In the DA strain, the reduced abundance of transcripts encoding ribosomal components (LSU-beta, 18S rRNA), MASP, and HSP70 is indicative of a coordinated remodeling of RNA steady-state levels that may ultimately impact protein synthesis capacity, stress adaptation, and immune evasion under drug pressure [52]. Rather than reflecting direct transcriptional inhibition, this pattern likely arises from altered mRNA stability, processing, or turnover mechanisms. In contrast, untreated DA controls showed higher levels of RHS proteins and UDP-GlcNAc pyrophosphorylase transcripts, consistent with basal requirements for genome plasticity and glyconjugate metabolism. The MG strain exhibited a broader and less targeted reduction in transcript abundance, encompassing GAPDH, trans-sialidases, and conserved hypothetical proteins—genes central to parasite metabolism, infectivity, and phenotypic flexibility [56,57]. Collectively, these findings point to a strain-specific remodeling of transcript abundance in response to Amiozole, reflecting differential post-transcriptional regulatory adaptations that affect metabolic pathways, ribosomal-associated functions, and surface architecture rather than direct modulation of the transcriptional machinery.

GO enrichment analysis revealed that Amiozole disrupts critical functional pathways in *T. cruzi*, with particularly pronounced effects in the DA strain. Notably, pathways related to ER–Golgi vesicular trafficking, intracellular localization, and pathogenesis were upregulated, suggesting a compensatory response aimed at redistributing proteins and metabolites to mitigate drug-induced cellular stress [58]. The formation of intra- and extracellular vesicles likely plays a central role in preserving homeostasis and coordinating adaptive mechanisms under these conditions. This aligns with the known effects of amiodarone, which perturbs calcium signaling and induces ER and mitochondrial dysfunction, leading to vesicle accumulation and collapse of secretory pathways [22]. Simultaneously, the downregulation of glycosylation and post-translational modification pathways implies a disruption in the processing of surface glycoproteins—molecules critical for immune evasion and host–parasite interactions [51]. Such impairment could limit the parasite’s adaptive capacity under nutritional or environmental stress, as observed during metacyclogenesis [54]. Together, these findings identify the secretory and glycosylation machinery as vulnerable nodes in *T. cruzi*’s stress response, positioning them as compelling therapeutic targets. Future functional validation through gene editing and subcellular organelle proteomics will be essential to assess the druggability of these systems and to inform strategies that exploit their fragility [59,60].

In the MG strain, Amiozole treatment triggered a selective activation of pathways involved in host–parasite interactions and glycoprotein glycosylation, indicative of a targeted effort to maintain surface architecture under pharmacological stress [39]. Simultaneously, the repression of processes such as intracellular transport, microtubule organization, and ion translocation suggests a substantial breakdown of cellular homeostasis. Given the centrality of microtubules to replication, division, motility, and vesicular trafficking, their disorganization likely compromises multiple essential functions critical for parasite survival [61]. This response mirrors the known effects of amiodarone on vesicular transport and calcium signaling, as well as itraconazole’s disruption of ergosterol biosynthesis and membrane stability [22], supporting the hypothesis that Amiozole exerts a synergistic impact on both structural and functional systems. To refine our understanding of its mechanism, future studies should combine high-resolution imaging, cytoskeletal integrity assays, and functional evaluations of motility, viability, and replication. Such approaches will be pivotal for characterizing Amiozole-induced subcellular alterations and for validating cytoskeletal destabilization as a viable therapeutic target in *T. cruzi*.

Metabolic pathway analysis revealed divergent transcriptomic responses to Amiozole in the DA (resistant) and MG (susceptible) *T. cruzi* strains, suggesting strain-specific adaptive strategies. Consistent with heterogeneous stress responses described in other protozoan parasites—such as *Toxoplasma gondii*, *Leishmania* spp., *Plasmodium falciparum*, and *T. brucei* [44,62–64]—the DA strain exhibited a restrained transcriptional activation, primarily involving purine metabolism, potentially as a compensatory mechanism to sustain vital functions [65]. In contrast, a broad repression of essential biosynthetic pathways was evident, particularly those governing the synthesis of complex lipids and structural glycoconjugates, including O-linked mucins, GPI anchors, and glycosphingolipids. This downregulation may impair surface remodeling processes critical for virulence and immune evasion [57]. To determine whether this transcriptional suppression translates into attenuated pathogenicity, future studies should investigate the structural integrity and functionality of the parasite surface post-treatment using immunolabeling, surface proteomics, and infectivity assays. Such approaches could unveil a therapeutic vulnerability in membrane renewal pathways, particularly relevant for overcoming drug resistance.

In the MG strain, Amiozole treatment induced a pronounced upregulation of sphingolipid metabolism, likely as a compensatory response to membrane damage or structural stress, consistent with observations in *T. cruzi* under metabolic challenge [48]. This activation, however, was accompanied by widespread repression of essential metabolic pathways, including oxidative phosphorylation, nucleotide metabolism (purines, pyrimidines, folate, riboflavin, thiamine), nitrogen metabolism, and taurine biosynthesis. Such a transcriptional landscape points to a profound metabolic collapse that could impair DNA integrity, organelle function, and ATP generation [65,66]. To confirm whether this dysregulation represents a functional tipping point, future studies should integrate assays of mitochondrial integrity, genomic stability, and replicative capacity. These efforts will be key to assessing whether metabolic failure constitutes a vulnerability that can be therapeutically exploited to potentiate Amiozole’s efficacy. Notably, recent research has shifted toward targeting core organelles—particularly the mitochondrion—via phenotypic screens and molecular approaches designed to disrupt energy homeostasis and genomic maintenance [67,68].

BNZ resistance in *T. cruzi* has been linked to a multifactorial molecular phenotype involving enhanced antioxidant defenses, alterations in DNA repair pathways, and impaired prodrug activation due to mutations in *TcNTR* [69,70]. Resistant strains frequently overexpress detoxification enzymes such as superoxide dismutases and glutathione transferases and display broad metabolic reprogramming that promotes survival under drug pressure [37,71]. Importantly, these features are not restricted to laboratory-selected lines but are increasingly documented in natural isolates from patients and vectors, underscoring their epidemiological relevance [14,70].

Comparative transcriptomic studies have consistently shown that BNZ resistance is accompanied by marked metabolic adjustments. For instance, Lima et al. (2023) reported strong overexpression of amino acid metabolism–related transcripts (GO:0006519), including kynureninases and aminotransferases, together with enrichment of ribosomal and translational processes (GO:0006412) [14]. In contrast, our analysis of naturally BNZ-resistant (DA) and -susceptible (MG) strains exposed to Amiozole reveals a distinct adaptive landscape. Although modulation of ribosomal genes was observed in both contexts, the Amiozole response was characterized by preferential activation of ER–Golgi trafficking, transmembrane export, and plasma membrane remodeling rather than dominant amino acid metabolic pathways. Notably, while BNZ-resistant strains typically show repression of redox and energy metabolism—affecting glycolytic enzymes and oxidative stress response proteins such as superoxide dismutases, peroxiredoxins, and thioredoxins—Amiozole treatment elicited a complex regulation of hydrolases, peptidases, and glycosylation-related processes [14,69]. This was particularly evident in the MG strain, where essential functions related to intracellular transport, cytoskeletal organization, and signaling were strongly repressed. Together, these differences suggest that BNZ resistance primarily reflects metabolic and redox adaptations aimed at drug detoxification, whereas Amiozole induces a broader reorganization of parasite–host interfaces, surface glycoprotein biogenesis (mucins, MASP, trans-sialidases), and membrane dynamics, highlighting adaptive strategies that extend beyond core energy metabolism.

Consistent with this distinction, Mejía et al. (2025) described a BNZ-resistant phenotype dominated by stress response and redox homeostasis pathways, with overexpression of heat shock proteins, hypoxia-induced proteins, CDC45, and glycosomal phosphoenolpyruvate carboxykinase, alongside enrichment of GO terms related to nucleobase metabolism, DNA replication and repair, and glutathione catabolism [15]. In contrast, our data indicate that Amiozole exposure does not primarily activate replication or canonical redox defense programs. Instead, it drives profound remodeling of ribosomal composition, vesicular trafficking, and cell surface architecture. Strikingly, whereas Mejía et al. reported consistent downregulation of ribosomal components (LSU-srRNA1/2, LSU-α, LSU-β) and mucin genes in BNZ-resistant strains, Amiozole treatment induced strong upregulation of ribosomal transcripts (18S rRNA, LSU-β) in the DA strain, suggesting a compensatory enhancement of translational capacity under pharmacological stress [54,55]. Moreover, although both studies identified modulation of energy metabolism and transmembrane transport, the functional direction diverged: BNZ resistance was associated with repression of mitochondrial transporters, TCA cycle activity, and lipid metabolism, whereas Amiozole exposure preferentially enriched ER–Golgi transport and hydrolase-mediated modification of surface glycoconjugates. In the MG strain, Amiozole additionally triggered extensive repression of intracellular transport, microtubule-based motility, and small-molecule binding, contrasting with activation of parasite–host interaction pathways and surface protein glycosylation. Collectively, these findings indicate that BNZ resistance relies on metabolic, redox, and replicative adaptations to genotoxic stress, whereas Amiozole induces a structurally and functionally broader remodeling centered on translational machinery, membrane architecture, and surface plasticity, supporting partially independent pharmacological adaptation mechanisms.

This distinction is further reinforced by comparison with Ospina et al. (2025), who reported that BNZ exposure in DA and MG strains predominantly upregulated genes associated with cytoskeletal dynamics, intracellular motility, and central metabolism, including kinesins, RHS proteins, trans-sialidases, and RAB6-like small GTPases, alongside coordinated activation of glycolysis, the pentose phosphate pathway, and the TCA cycle [38]. Such profiles suggest that BNZ tolerance depends on sustaining ATP production, reducing power, and biosynthetic precursors. In contrast, Amiozole-treated strains did not exhibit generalized activation of central metabolic pathways. Instead, the DA strain displayed significant repression of carbohydrate and lipid metabolism, including sphingolipid and fructose/mannose pathways and multiple routes of glycoconjugate biosynthesis (mucins, GPI anchors, glycosphingolipids), with selective activation of purine metabolism [48]. While both studies identified modulation of RHS and surface-associated genes, their functional context differed substantially: under BNZ pressure these genes aligned with a metabolically active resistant state, whereas under Amiozole they coincided with enrichment of ER–Golgi transport, hydrolase activity, and parasite–host interaction processes. In the MG strain, Amiozole induced an opposing pattern to BNZ exposure, marked by activation of sphingolipid metabolism and coordinated repression of essential energetic and biosynthetic pathways, indicative of metabolic compromise.

Collectively, the findings indicate that Amiozole exerts a disruptive effect on the central metabolism of *T. cruzi*, eliciting a more coordinated response in the resistant DA strain—likely linked to adaptive mechanisms—while inducing profound dysregulation in the susceptible MG strain. TEM revealed marked ultrastructural alterations post-treatment, including autophagosome formation, membrane vesiculation, Golgi disorganization, and kinetoplast decompaction in DA, consistent with reticular stress and activation of autophagy. Similar cytoplasmic damage patterns have been reported with amiodarone in *T. cruzi* and other protozoan models, such as *Trichomonas vaginalis* [23,72]. These structural observations are in line with transcriptomic profiles showing activation of protein processing and autophagy pathways, coupled with repression of lipid and glycoconjugate biosynthesis. The accumulation of vesicles and damage to reservosomes and the Golgi apparatus suggest an ongoing intracellular reorganization in response to pharmacological stress [17]. Notably, similar ultrastructural effects—such as mitochondrial swelling, autophagic vacuole formation, and expansion of acidocalcisomes—have been observed in *L.amazonensis* treated with sterol biosynthesis inhibitors (SBIs) like ketoconazole, reinforcing the notion of conserved stress responses across kinetoplastids [73].

In the MG strain, Amiozole induced extensive ultrastructural damage, including swelling and disintegration of the perinuclear space, kinetoplast disruption, detachment of reservosomes, autophagosome formation, and vesicle accumulation within the flagellar pocket, accompanied by abnormal projections along the plasma membrane. These alterations closely resemble those described by Chaves *et al*. (2022) [23], who reported irreversible cytoskeletal remodeling and apoptotic features—such as vesiculation and cytoplasmic retraction in amastigotes—via TEM. The structural collapse observed here is consistent with the marked transcriptomic repression of essential pathways, including energy metabolism and intracellular transport. Similar cytopathic effects have been associated with the Amiodarone–Ravuconazole combination, which exerts potent trypanocidal activity against amastigotes [74]. Collectively, these findings confirm that Amiozole imposes severe cellular stress, undermining both structural integrity and parasite viability. A comparable pattern has also been reported for heterocyclic agents such as Fexinidazole—approved for African trypanosomiasis—which induces extensive disorganization of reservosomes and disrupts the mitochondrion–kinetoplast complex [75].

A clear correspondence between the observed ultrastructural damage and transcriptional changes supports a synergistic mechanism of action for Amiozole, consistent with drug-induced stress responses reported in other parasitic systems, including *Schistosoma mansoni* [36]. Although our analyses were restricted to EPs, a non-infective stage, similar cellular pathways—such as mitochondrial dysfunction, membrane remodeling, and stress-response signaling—have been shown to be conserved across *T. cruzi* life stages and are also implicated in benznidazole-induced damage in intracellular amastigotes and preclinical infection models [76]. In this context, the adaptive remodeling observed in the naturally less susceptible DA strain may reflect compensatory mechanisms that enable partial survival under drug pressure, whereas the extensive structural and physiological collapse detected in the MG strain is indicative of reduced cellular resilience, a pattern analogous to benznidazole-susceptible phenotypes described in mammalian-stage parasites [15,24,71]. While direct extrapolation to infective stages must be approached with caution, these parallels suggest that the pathways perturbed by Amiozole in EPs may be relevant to therapeutically important stages [23,27,30,48]. Future studies incorporating amastigotes, *in vivo* infection models, and integrative multi-omic analyses—including proteomics and metabolomics—will be essential to validate these findings and to further define the translational potential of Amiozole as a candidate therapy for Chagas disease.

Our findings reveal a strain-specific response to Amiozole, where the divergence between DA and MG precluded the identification of a unified mechanism of action and instead highlighted distinct effects on each strain’s biology. This outcome, far from unexpected, aligns with the remarkable genomic plasticity of *T. cruzi*, which enables adaptation to adverse conditions through the differential activation of large multigene families linked to drug resistance [77]. Such adaptability is underpinned by mechanisms including aneuploidy, chromosomal rearrangements, and copy number variation (CNV), all of which can dynamically modulate gene expression and confer selective advantages under pharmacological pressure. Moreover, the potential involvement of epigenetic mechanisms—such as histone modifications, non-coding RNAs, and chromatin remodeling—cannot be ruled out, as these may contribute to a flexible and adaptive transcriptional response, particularly upon exposure to compounds like Amiozole [78]. In this context, future studies should incorporate single-cell transcriptomics and comparative epigenomic profiling to map functional and epigenetic heterogeneity at the population level, which is crucial for understanding *T. cruzi* persistence and therapeutic evasion [40,79].

Compared with studies based on *in vitro*–selected BNZ-resistant lines, our work offers a complementary perspective by jointly examining transcriptomic responses and ultrastructural alterations induced by Amiozole in naturally divergent *T. cruzi* strains. Nevertheless, several limitations should be considered when interpreting these findings. Transcriptomic analyses were restricted to EPs, a non-infective life stage, limiting direct extrapolation to host-relevant mechanisms. In addition, only two genetically contrasting strains were evaluated, which, while informative for strain-specific responses, constrains broader generalization across the extensive genetic diversity of *T. cruzi*. The use of a single drug concentration precluded assessment of dose-dependent effects, and the lack of complete genome assemblies limited the identification of structural variants or regulatory mutations influencing gene expression. Furthermore, *in vitro* selection of Amiozole-resistant strains was not performed, as this study focused on acute responses to short-term drug exposure; therefore, the potential emergence of adaptive resistance under prolonged or stepwise treatment cannot be excluded. Finally, differential responses to Amiozole may reflect strain-specific differences in basal metabolism, replication dynamics, transcriptional regulation, or epigenetic mechanisms not addressed here. Future studies integrating multi-omic approaches, infective life stages, broader strain panels, and long-term drug selection will be essential to better define genotype–phenotype–drug response relationships and refine therapeutic strategies.

## Conclusions

The combined evaluation of transcriptomic and ultrastructural responses in BNZ-resistant (DA) and -sensitive (MG) *T. cruzi* strains following Amiozole exposure reveals a complex pattern of cellular adaptation and damage. This pattern is characterized by the selective activation of metabolic pathways and cellular defense mechanisms in the resistant strain, alongside a pronounced suppression of essential biosynthetic and energetic pathways in both strains. These findings suggest that Amiozole induces profound metabolic and structural stress that compromises parasite viability, supporting its potential use in synergistic treatment strategies to overcome the current challenge of drug resistance.

Although our study provides a novel perspective by combining transcriptomic and ultrastructural analyses, several limitations must be considered. These include the exclusive use of epimastigote forms, the evaluation of only two strains, and the assessment at a single drug concentration, which together constrain the extrapolation and comprehensive understanding of the molecular mechanisms underlying Amiozole’s mode of action. Therefore, future studies incorporating different developmental stages, a broader range of strains accounting for intra-DTU variability, dose-response analyses, and complete genomic assessments will be essential to validate and further elucidate Amiozole’s therapeutic potential in treating CD.

## Supporting information

S1 Fig *In vitro* parasite viability curves and corresponding IC_50_ values for *Trypanosoma cruzi* DA and MG strains following exposure to benznidazole (A–B) and the combined amiodarone–itraconazole treatment (Amiozole) (C–D). (PNG)

S1 Table Differentially expressed genes in the DA Strain: Genes with the highest and lowest Log2FoldChange. (DOCX)

S2 Table Differentially expressed genes in the MG Strain: Genes with the highest and lowest Log2FoldChange. (DOCX)

S3 Table Gene Ontology (GO) enrichment analysis for the DA strain, showing significantly enriched terms across the three GO categories: Biological Process (BP), Cellular Component (CC), and Molecular Function (MF). (DOCX)

S4 Table Gene Ontology (GO) enrichment analysis for the MG strain, showing significantly enriched terms across the three GO categories: Biological Process (BP), Cellular Component (CC), and Molecular Function (MF). (DOCX)
