# Correction: Transcriptomic and ultrastructural responses to Amiodarone–Itraconazole in naturally benznidazole-resistant and -susceptible *Trypanosoma cruzi* strains

**DOI:** 10.1371/journal.pntd.0014328

**Published:** 2026-05-20

**Authors:** Stivenn Gutiérrez, Carlos Ospina, Tatiana Cáceres, Luz Helena Patiño, Alberto Paniz-Mondolfi, Juan David Ramírez

The following information is missing from the Funding statement: Sequencing costs were funded by internal departmental funds from the Department of Pathology, Molecular and Cell-Based Medicine, Icahn School of Medicine at Mount Sinai.
