# Supplementary figures and images for: Transcriptomic and ultrastructural responses to Amiodarone–Itraconazole in naturally benznidazole-resistant and -susceptible Trypanosoma cruzi strains
Source: PLoS Negl Trop Dis. 2026 Jan 14;20(1):e0013916. doi: 10.1371/journal.pntd.0013916 (PMC12863684; doi:10.1371/journal.pntd.0013916)

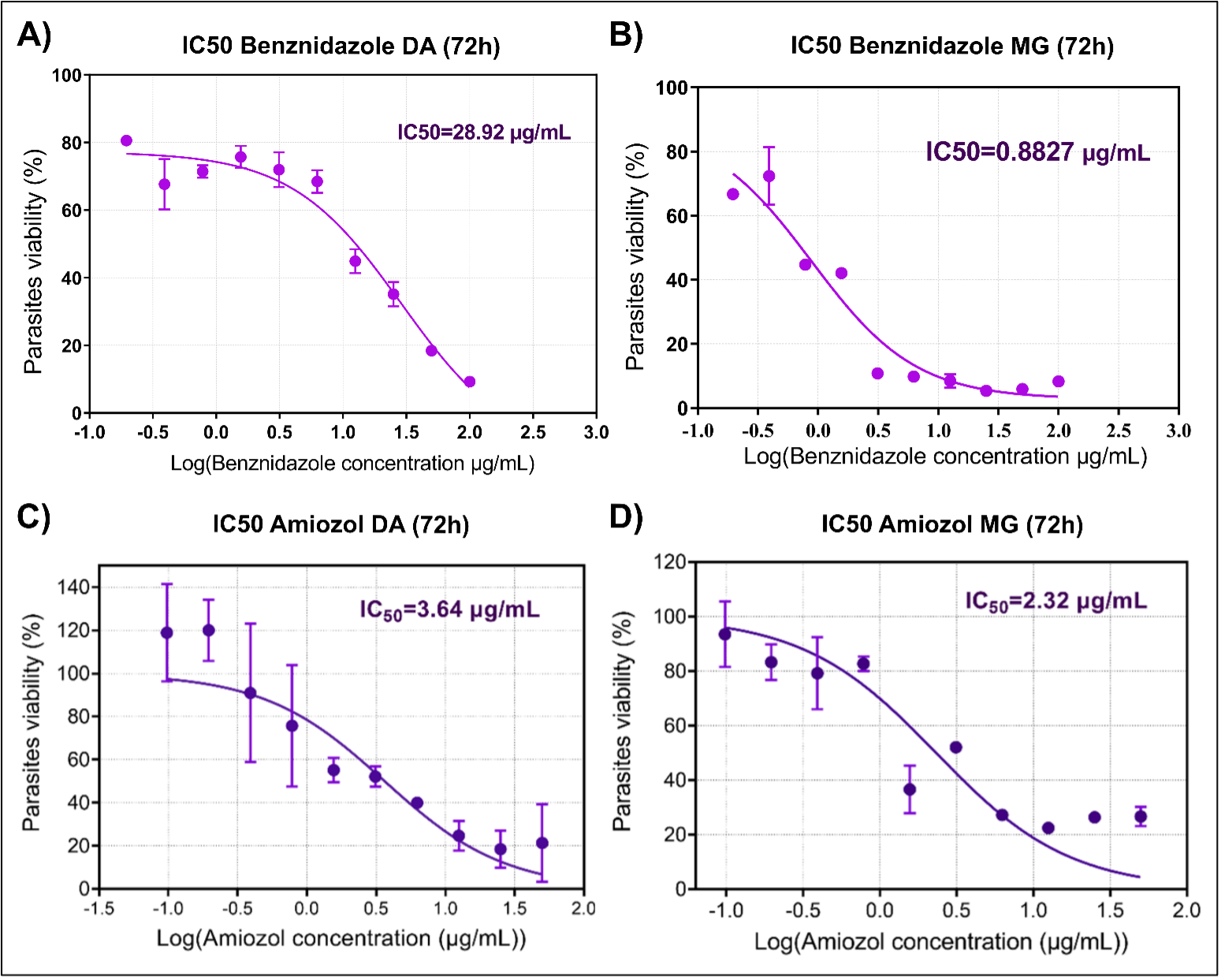

Supplement: S1 Fig — (PNG) [file pntd.0013916.s001.png]
