# Supplementary material for: Transcriptomic and ultrastructural responses to Amiodarone–Itraconazole in naturally benznidazole-resistant and -susceptible Trypanosoma cruzi strains
Source: PLoS Negl Trop Dis. 2026 Jan 14;20(1):e0013916. doi: 10.1371/journal.pntd.0013916 (PMC12863684; doi:10.1371/journal.pntd.0013916)
Supplement: S1 Table — (DOCX) [file pntd.0013916.s002.docx]

**S1 Table.** Differentially expressed genes in the DA Strain: Genes with the highest and lowest Log2FoldChange.

| **Gene ID** | **Log2FoldChange** | **LfcSE** | **Pvalue** | **Description** |
| --- | --- | --- | --- | --- |
| **UP-REGULATED GENES** | | | | |
| C4B63_118g10 | 1.539302501 | 0.1850786 | 9.02E-17 | trans-sialidase, Group II |
| C4B63_133g58 | 3.566270345 | 1.0051585 | 0.0003882 | heat shock protein 70 (hsp70) |
| C4B63_16g175 | 1.626851681 | 0.2021328 | 8.386E-16 | conserved hypothetical protein |
| C4B63_16g69 | 1.536651244 | 0.6443944 | 0.0170957 | unspecified product |
| C4B63_170g37 | 2.298537779 | 0.8769527 | 0.0087659 | conserved hypothetical protein |
| C4B63_190nc21 | 1.733727043 | 0.8593759 | 0.0436511 | Large subunit ribosomal RNA,note=Large subunit ribosomal RNA, LSU-beta, (old nomenclature, 24S-beta) |
| C4B63_190nc3 | 1.523124669 | 0.7604672 | 0.0451902 | 18S ribosomal RNA,note=SSU |
| C4B63_190nc31 | 2.174672921 | 0.5238478 | 3.305E-05 | Large subunit ribosomal RNA, LSU-srRNA4,note=Old nomenclature 24S-S4 |
| C4B63_190nc37 | 1.714903913 | 0.6476452 | 0.0080992 | Large subunit ribosomal RNA,note=Large subunit ribosomal RNA, LSU-beta, (old nomenclature, 24S-beta) |
| C4B63_22nc11 | 1.573806949 | 0.6663729 | 0.0181889 | Large subunit ribosomal RNA,note=Large subunit ribosomal RNA, LSU-beta, (old nomenclature, 24S-beta) |
| C4B63_232g5 | 2.455349871 | 0.8404205 | 0.0034827 | unspecified product |
| C4B63_235g8c | 1.980169316 | 0.9204601 | 0.0314539 | conserved hypothetical protein |
| C4B63_240g5 | 1.640543711 | 0.2686147 | 1.013E-09 | trans-sialidase, Group II |
| C4B63_258g24 | 2.289258384 | 0.971356 | 0.0184349 | expression site-associated gene (ESAG-like) protein |
| C4B63_267g6 | 1.834240571 | 0.7176776 | 0.0105944 | protein transport protein Sec24C(fragment) |
| C4B63_27g271 | 1.575556196 | 0.5206907 | 0.002479 | unspecified product |
| C4B63_2nc182 | 4.719867244 | 1.2362321 | 0.0001346 | unspecified product |
| C4B63_335nc8 | 2.461333063 | 1.1485334 | 0.0321113 | Large subunit ribosomal RNA,note=Large subunit ribosomal RNA, LSU-beta, (old nomenclature, 24S-beta) |
| C4B63_356g5 | 1.735943607 | 0.8368499 | 0.0380441 | unspecified product |
| C4B63_476nc1 | 4.48581041 | 1.5385412 | 0.0035498 | 18S ribosomal RNA,note=SSU |
| C4B63_476nc7 | 2.322754578 | 1.1353921 | 0.0407787 | Large subunit ribosomal RNA,note=Large subunit ribosomal RNA, LSU-alpha, (old nomenclature, 24S-alpha) |
| C4B63_476nc8 | 1.678106979 | 0.7511219 | 0.0254743 | Large subunit ribosomal RNA, LSU-srRNA1,note=Old nomenclature 24S-S1 |
| C4B63_476nc9 | 3.507066174 | 1.2712472 | 0.0058021 | Large subunit ribosomal RNA,note=Large subunit ribosomal RNA, LSU-beta, (old nomenclature, 24S-beta) |
| C4B63_495nc7 | 3.012550234 | 1.0148191 | 0.002992 | Large subunit ribosomal RNA, LSU-srRNA4,note=Old nomenclature 24S-S4 |
| C4B63_4g381 | 1.50719054 | 0.7234937 | 0.0372319 | transporter |
| C4B63_511nc3 | 2.787472161 | 1.1182281 | 0.0126755 | 18S ribosomal RNA,note=SSU |
| C4B63_511nc7 | 3.825744634 | 1.3075256 | 0.0034341 | Large subunit ribosomal RNA,note=Large subunit ribosomal RNA, LSU-beta, (old nomenclature, 24S-beta) |
| C4B63_530nc8 | 2.605404412 | 0.8035582 | 0.0011855 | Large subunit ribosomal RNA,note=Large subunit ribosomal RNA, LSU-beta, (old nomenclature, 24S-beta) |
| C4B63_577nc1 | 4.274300149 | 1.3684942 | 0.001788 | 18S ribosomal RNA,note=SSU |
| C4B63_69nc1 | 2.220181779 | 0.914819 | 0.0152281 | H/ACA snoRNA,ncRNA class=snoRNA |
| C4B63_71g114 | 1.537585611 | 0.5771156 | 0.0077158 | mucin TcMUCII |
| C4B63_72g19 | 1.640757721 | 0.7492018 | 0.0285237 | trans-sialidase, Group II |
| C4B63_73g13 | 2.165798398 | 1.0996064 | 0.0488828 | unspecified product |
| C4B63_96g29 | 1.903270475 | 0.9589441 | 0.0471716 | unspecified product |
| C4B63_97g34 | 2.937784275 | 1.11672 | 0.0085203 | P-type H+-ATPase |
| **DOWN-REGULATED GENES** | | | | |
| C4B63_100g37 | -2.398896922 | 0.7938748 | 0.0025131 | Mucin-associated surface protein (MASP) |
| C4B63_104g70 | -2.330235066 | 0.847468 | 0.005966 | UDP-Gal or UDP-GlcNAc-dependent glycosyltransferase |
| C4B63_107g46 | -1.529167006 | 0.4272225 | 0.0003445 | Mucin-associated surface protein (MASP) |
| C4B63_107g97 | -1.623165183 | 0.5572641 | 0.0035827 | unspecified product |
| C4B63_119g22 | -1.518626739 | 0.767988 | 0.0479954 | retrotransposon hot spot protein (RHS) |
| C4B63_11g121 | -1.539355187 | 0.7608838 | 0.0430613 | Mucin-associated surface protein (MASP) |
| C4B63_11g127 | -2.393190408 | 1.179756 | 0.0425045 | unspecified product |
| C4B63_11g276 | -1.533387631 | 0.6756808 | 0.0232442 | Mucin-associated surface protein (MASP) |
| C4B63_11g351 | -2.361236845 | 0.9381049 | 0.0118349 | Mucin-associated surface protein (MASP) |
| C4B63_129g6 | -1.666179443 | 0.7322909 | 0.0228881 | retrotransposon hot spot (RHS) protein |
| C4B63_12g104 | -1.806131788 | 0.3889169 | 3.417E-06 | Mucin-associated surface protein (MASP) |
| C4B63_12g58 | -2.037144461 | 0.8888764 | 0.0219161 | unspecified product |
| C4B63_130g20 | -2.351559067 | 1.0847887 | 0.0301771 | unspecified product |
| C4B63_130g28 | -1.799671745 | 0.5553776 | 0.0011934 | L1Tc protein Reverse transcriptase/Endonuclase/Rnase H domains |
| C4B63_138g21 | -1.724499582 | 0.5068598 | 0.0006682 | elongation factor 1-gamma (EF-1-gamma) |
| C4B63_13g113 | -1.509857934 | 0.3632614 | 3.233E-05 | zinc-finger of a C2HC-type |
| C4B63_141g4 | -1.510136615 | 0.6538731 | 0.0209144 | unspecified product |
| C4B63_147g39 | -2.037090979 | 0.8434796 | 0.0157307 | ADP-ribosylation factor family |
| C4B63_150g15 | -1.578839552 | 0.5536861 | 0.0043513 | unspecified product |
| C4B63_15g7 | -2.029182167 | 0.7141928 | 0.0044941 | protein kinase |
| C4B63_17g327 | -2.599312891 | 0.773958 | 0.0007838 | retrotransposon hot spot (RHS) protein |
| C4B63_191g31 | -1.645207188 | 0.5009 | 0.0010216 | protein kinase |
| C4B63_1g1090 | -1.721163683 | 0.3499169 | 8.709E-07 | unspecified product |
| C4B63_1g1232 | -1.951510521 | 0.7337683 | 0.007824 | unspecified product |
| C4B63_1g244 | -1.682896818 | 0.8204867 | 0.0402576 | Mucin-associated surface protein (MASP) |
| C4B63_1g389 | -1.598708792 | 0.735997 | 0.029843 | retrotransposon hot spot protein (RHS) |
| C4B63_1g434 | -1.52876325 | 0.314674 | 1.184E-06 | mucin TcMUCII |
| C4B63_1g626 | -1.79792212 | 0.6146068 | 0.003441 | unspecified product |
| C4B63_1g784 | -1.54112702 | 0.546449 | 0.0047985 | unspecified product |
| C4B63_1g811 | -1.762554401 | 0.7802937 | 0.0238937 | unspecified product |
| C4B63_214g20 | -3.533096943 | 1.0628544 | 0.0008869 | Sphingosine N-acyltransferase |
| C4B63_255g21 | -2.248682777 | 0.9654478 | 0.0198506 | calreticulin |
| C4B63_279g11 | -1.628890551 | 0.2864293 | 1.294E-08 | alpha tubulin |
| C4B63_287g6 | -1.582350431 | 0.6974484 | 0.0232823 | SLACS reverse transcriptase |
| C4B63_290g9 | -1.503915075 | 0.6768354 | 0.0262846 | unspecified product |
| C4B63_29g171 | -1.584088612 | 0.7401316 | 0.0323322 | unspecified product |
| C4B63_2nc143 | -2.20016631 | 1.0970063 | 0.0448979 | C/D small nucleolar RNA (snoRNA),ncRNA class=snoRNA |
| C4B63_319g4 | -2.147229085 | 0.8485804 | 0.011394 | conserved hypothetical protein |
| C4B63_31g159 | -1.655829399 | 0.7400533 | 0.0252572 | unspecified product |
| C4B63_321g18 | -2.016884598 | 0.955216 | 0.0347342 | UDP-Gal or UDP-GlcNAc-dependent glycosyltransferase |
| C4B63_33g275 | -1.655833831 | 0.7688453 | 0.0312666 | Mucin-associated surface protein (MASP) |
| C4B63_33g57 | -1.6251717 | 0.6459287 | 0.0118687 | unspecified product |
| C4B63_36g18 | -1.766583589 | 0.6222122 | 0.0045227 | mucin TcMUCII |
| C4B63_36g373 | -2.023105736 | 0.8178558 | 0.0133733 | unspecified product |
| C4B63_36g86 | -1.87656002 | 0.734017 | 0.0105712 | Mucin-associated surface protein (MASP) |
| C4B63_37g395 | -2.052537378 | 0.932015 | 0.0276471 | retrotransposon hot spot protein (RHS) |
| C4B63_389g6 | -1.573757825 | 0.4339521 | 0.0002872 | SLACS reverse transcriptase |
| C4B63_38g259 | -5.928212798 | 1.5785834 | 0.0001731 | UDP-Gal or UDP-GlcNAc-dependent glycosyltransferase |
| C4B63_39g235 | -2.139775184 | 0.8551717 | 0.0123439 | conserved hypothetical protein |
| C4B63_39g87 | -2.026478291 | 0.8532128 | 0.0175435 | Mucin-associated surface protein (MASP) |
| C4B63_3g110 | -1.803099576 | 0.6826851 | 0.0082616 | unspecified product |
| C4B63_3g405 | -2.26004658 | 1.107904 | 0.0413573 | trans-sialidase, Group V |
| C4B63_3g447 | -2.031368344 | 0.8802766 | 0.0210187 | mucin-associated surface protein (MASP) |
| C4B63_3g501 | -1.540961489 | 0.7251687 | 0.0335891 | trans-sialidase, Group VI |
| C4B63_3g71 | -1.572867942 | 0.7808778 | 0.0439853 | conserved hypothetical protein |
| C4B63_3g733 | -3.035180191 | 1.3071184 | 0.0202308 | Mucin-associated surface protein (MASP) |
| C4B63_405g11 | -1.528685546 | 0.6441616 | 0.0176376 | retrotransposon hot spot (RHS) protein |
| C4B63_4g225 | -1.520706313 | 0.4591762 | 0.0009269 | conserved hypothetical protein |
| C4B63_504g2 | -2.609291155 | 1.030302 | 0.0113236 | unspecified product |
| C4B63_50g134 | -1.731376195 | 0.494202 | 0.0004594 | retrotransposon hot spot protein (RHS) |
| C4B63_53g211 | -1.878900282 | 0.5656701 | 0.0008952 | conserved hypothetical protein |
| C4B63_55g55 | -1.576720356 | 0.7469282 | 0.0347775 | Paraflagellar rod protein 2 |
| C4B63_55g65 | -1.795433076 | 0.6608002 | 0.0065865 | ADP-ribosylation factor |
| C4B63_59g103 | -1.667750343 | 0.2909279 | 9.895E-09 | surface protease GP63 |
| C4B63_5g123 | -1.538603595 | 0.4948372 | 0.0018752 | Mucin-associated surface protein (MASP) |
| C4B63_5g194 | -1.564010998 | 0.5689235 | 0.0059764 | unspecified product |
| C4B63_5g210 | -1.795917326 | 0.7638863 | 0.0187217 | unspecified product |
| C4B63_5g296 | -1.603936855 | 0.7990275 | 0.0447112 | mucin TcMUCII |
| C4B63_5g372 | -1.567980314 | 0.4351056 | 0.0003137 | Mucin-associated surface protein (MASP) |
| C4B63_5g395 | -2.651327453 | 0.6382451 | 3.266E-05 | retrotransposon hot spot protein (RHS) |
| C4B63_62g182 | -2.080298886 | 0.6750636 | 0.0020587 | Mucin-associated surface protein (MASP) |
| C4B63_64g119 | -1.858637942 | 0.7092199 | 0.0087755 | unspecified product |
| C4B63_64g124 | -1.550046093 | 0.6854556 | 0.0237384 | mucin TcMUCII |
| C4B63_69g164 | -1.841993571 | 0.5657422 | 0.0011304 | unspecified product |
| C4B63_6g400 | -1.777436947 | 0.8362659 | 0.0335495 | conserved hypothetical protein |
| C4B63_71g48 | -2.285958123 | 1.1034958 | 0.0383065 | conserved hypothetical protein |
| C4B63_74g118 | -1.787895969 | 0.6266175 | 0.0043275 | unspecified product |
| C4B63_74g13 | -2.329032958 | 0.8349618 | 0.0052808 | Mucin-associated surface protein (MASP) |
| C4B63_74g17 | -2.234557905 | 0.6770987 | 0.0009662 | Mucin-associated surface protein (MASP) |
| C4B63_7g207 | -1.612309603 | 0.5416698 | 0.0029151 | antigenic protein |
| C4B63_80g51 | -1.63776388 | 0.3697893 | 9.471E-06 | Transport protein particle subunit trs23 |
| C4B63_88g84 | -2.19215578 | 0.8486707 | 0.0097932 | unspecified product |
| C4B63_8g233 | -1.669669595 | 0.5375894 | 0.0018974 | unspecified product |
| C4B63_8g258 | -2.465527088 | 0.8229856 | 0.002737 | mucin TcMUCII |
| C4B63_90g3 | -2.053298123 | 0.5307756 | 0.0001095 | mucin TcMUCII |
| C4B63_90g43 | -2.338751946 | 1.1290037 | 0.0383104 | conserved hypothetical protein |
| C4B63_92g84 | -1.957016492 | 0.6181706 | 0.0015465 | SLACS reverse transcriptase (fragment) |
