# Supplementary material for: Transcriptomic and ultrastructural responses to Amiodarone–Itraconazole in naturally benznidazole-resistant and -susceptible Trypanosoma cruzi strains
Source: PLoS Negl Trop Dis. 2026 Jan 14;20(1):e0013916. doi: 10.1371/journal.pntd.0013916 (PMC12863684; doi:10.1371/journal.pntd.0013916)
Supplement: S2 Table — (DOCX) [file pntd.0013916.s003.docx]

**S2 Table.** Differentially expressed genes in the MG Strain: Genes with the highest and lowest Log2FoldChange.

| **Gene ID** | **Log2FoldChange** | **LfcSE** | **Pvalue** | **Description** |
| --- | --- | --- | --- | --- |
| **UP-REGULATED GENES** | | | | |
| C4B63_104g70 | 1.810703008 | 0.8951239 | 0.0430884 | UDP-Gal or UDP-GlcNAc-dependent glycosyltransferase |
| C4B63_118g10 | 1.867276258 | 0.1643131 | 6.308E-30 | trans-sialidase, Group II |
| C4B63_11g115 | 1.57439207 | 0.4255273 | 0.0002157 | unspecified product |
| C4B63_11g429 | 1.592278435 | 0.5453477 | 0.0035031 | unspecified product |
| C4B63_121g66 | 1.52160975 | 0.5350123 | 0.0044542 | mucin TcMUCII |
| C4B63_123g76 | 1.650000629 | 0.7307591 | 0.0239502 | mucin TcMUCII |
| C4B63_126g18 | 1.872599302 | 0.7414663 | 0.0115522 | unspecified product |
| C4B63_12g181 | 2.003023828 | 0.6339795 | 0.0015807 | conserved hypothetical protein |
| C4B63_135g28 | 1.789950628 | 0.5512796 | 0.0011667 | unspecified product |
| C4B63_13g332 | 2.691823453 | 0.789676 | 0.0006526 | RNA-binding protein |
| C4B63_151g46 | 3.161238618 | 1.2638692 | 0.012376 | unspecified product |
| C4B63_172g21 | 1.752255775 | 0.8636319 | 0.0424646 | unspecified product |
| C4B63_18g1166c | 2.266193635 | 0.9187752 | 0.0136426 | conserved hypothetical protein |
| C4B63_190nc31 | 1.871410975 | 0.5574478 | 0.0007876 | Large subunit ribosomal RNA, LSU-srRNA4,note=Old nomenclature 24S-S4 |
| C4B63_1g1004 | 1.872471535 | 0.5652171 | 0.0009236 | Mucin-associated surface protein (MASP) |
| C4B63_1g1397 | 1.90073847 | 0.2301335 | 1.465E-16 | trans-sialidase, Group II |
| C4B63_204nc1 | 2.342048905 | 0.8582357 | 0.0063544 | 5S ribosomal RNA |
| C4B63_209g8 | 1.795828488 | 0.1423287 | 1.692E-36 | trans-sialidase, Group II |
| C4B63_216g12 | 2.758478015 | 0.3375754 | 3.047E-16 | protein associated with differentiation 4 |
| C4B63_216g7 | 2.616847657 | 0.8730279 | 0.0027226 | protein associated with differentiation 4 |
| C4B63_21g185 | 2.033309424 | 0.2942355 | 4.83E-12 | unspecified product |
| C4B63_21g272 | 1.639816617 | 0.7740324 | 0.0341296 | conserved hypothetical protein |
| C4B63_240g3 | 1.557261941 | 0.4386565 | 0.0003851 | trans-sialidase, Group II |
| C4B63_240g5 | 1.780317995 | 0.1773188 | 1.015E-23 | trans-sialidase, Group II |
| C4B63_24g260 | 1.65138429 | 0.5570654 | 0.0030324 | Mucin-associated surface protein (MASP) |
| C4B63_276g4 | 1.625849534 | 0.4028431 | 5.438E-05 | unspecified product |
| C4B63_279g15 | 1.943173902 | 0.7889024 | 0.0137728 | unspecified product |
| C4B63_282g20 | 1.622500796 | 0.3444338 | 2.469E-06 | surface protease GP63 |
| C4B63_29g330 | 1.719177116 | 0.7477002 | 0.0214887 | Mucin-associated surface protein (MASP) |
| C4B63_29g369 | 2.23118047 | 1.0391526 | 0.0317841 | conserved hypothetical protein |
| C4B63_2g453 | 1.58543488 | 0.1042676 | 3.254E-52 | conserved hypothetical protein |
| C4B63_2g4640c | 2.31722326 | 0.9310847 | 0.0128198 | conserved hypothetical protein |
| C4B63_30g255 | 2.773495224 | 1.2116078 | 0.0220734 | surface protease GP63 |
| C4B63_32g172 | 2.338488464 | 0.8361868 | 0.0051642 | trans-sialidase, Group II |
| C4B63_32nc3 | 1.689149652 | 0.6245946 | 0.0068429 | tRNA-Ile |
| C4B63_33g305 | 2.34663721 | 0.8342322 | 0.0049092 | mucin TcMUCII |
| C4B63_341g9 | 1.701912421 | 0.450525 | 0.0001583 | surface protease GP63 |
| C4B63_36g169 | 1.840601391 | 0.5187283 | 0.0003877 | mucin TcMUCII |
| C4B63_38g189 | 1.808694345 | 0.7729702 | 0.0192875 | trans-sialidase, Group II |
| C4B63_399g10 | 1.871635658 | 0.2221582 | 3.614E-17 | unspecified product |
| C4B63_3g672 | 1.686479659 | 0.3948355 | 1.943E-05 | unspecified product |
| C4B63_3g733 | 1.879777847 | 0.7963446 | 0.0182499 | Mucin-associated surface protein (MASP) |
| C4B63_430g6 | 2.228862405 | 0.5319928 | 2.794E-05 | UDP-Gal or UDP-GlcNAc-dependent glycosyltransferase |
| C4B63_50g234 | 2.254850724 | 0.6883942 | 0.0010547 | Endonuclease-reverse transcriptase(fragment) |
| C4B63_51g222 | 1.902416397 | 0.5244259 | 0.000286 | unspecified product |
| C4B63_60g197 | 1.682562273 | 0.600513 | 0.0050807 | retrotransposon hot spot (RHS) protein |
| C4B63_62g154 | 1.95979072 | 0.9605203 | 0.0413162 | unspecified product |
| C4B63_62g18 | 1.617047617 | 0.7697233 | 0.0356571 | Mucin-associated surface protein (MASP) |
| C4B63_62g22 | 2.077709933 | 0.6136959 | 0.0007103 | mucin TcMUCII |
| C4B63_64g125 | 1.559179746 | 0.7882392 | 0.0479226 | unspecified product |
| C4B63_67g14 | 1.822423587 | 0.6469994 | 0.0048515 | unspecified product |
| C4B63_68g103 | 1.620210485 | 0.2430288 | 2.615E-11 | trans-sialidase, Group II |
| C4B63_72g6 | 1.74567886 | 0.1997282 | 2.325E-18 | trans-sialidase, Group II |
| C4B63_74g95 | 1.96614516 | 0.598989 | 0.0010291 | retrotransposon hot spot protein (RHS) |
| C4B63_83g37 | 1.828570455 | 0.1953481 | 7.93E-21 | unspecified product |
| C4B63_92g50 | 2.199337327 | 0.7574168 | 0.0036874 | conserved hypothetical protein |
| C4B63_96g87 | 1.932187323 | 0.8032259 | 0.0161488 | unspecified product |
| **DOWN-REGULATED GENES** | | | | |
| C4B63_100g27 | -2.150526115 | 1.01981 | 0.034966 | unspecified product |
| C4B63_103g75 | -1.647812669 | 0.6601512 | 0.0125562 | trans-sialidase, Group VI |
| C4B63_105g54 | -1.528299534 | 0.5454538 | 0.0050805 | retrotransposon hot spot (RHS) protein |
| C4B63_108g13 | -1.813519424 | 0.7748825 | 0.0192641 | retrotransposon hot spot protein (RHS) |
| C4B63_108g15 | -1.637053558 | 0.4018602 | 4.627E-05 | retrotransposon hot spot (RHS) protein |
| C4B63_10g154 | -1.655316462 | 0.3714414 | 8.332E-06 | RNA-binding protein |
| C4B63_10g293 | -1.510971566 | 0.391623 | 0.0001142 | Phosphatidylinositol 4-phosphate 5-kinase 6 |
| C4B63_10g439 | -1.696902359 | 0.1924008 | 1.148E-18 | heat shock protein 90 |
| C4B63_10g453 | -1.570263289 | 0.5429729 | 0.0038283 | ABC transporter |
| C4B63_10g454 | -1.939367118 | 0.4477026 | 1.479E-05 | ABC transporter(fragment) |
| C4B63_10g455 | -1.836666714 | 0.6989135 | 0.0085917 | ABC transporter |
| C4B63_10g472 | -2.019213069 | 0.4102953 | 8.594E-07 | conserved hypothetical protein |
| C4B63_10g490 | -1.573987438 | 0.1340741 | 7.979E-32 | Hsc70-interacting protein (Hip) |
| C4B63_10g512 | -2.40372859 | 0.4553763 | 1.302E-07 | membrane associated protein |
| C4B63_10g513 | -1.672474346 | 0.4143157 | 5.42E-05 | membrane associated protein |
| C4B63_110g37 | -1.56168828 | 0.4063384 | 0.0001214 | multidrug resistance protein E |
| C4B63_113g30 | -2.311590189 | 0.4797838 | 1.45E-06 | heat shock protein 85 |
| C4B63_114g35 | -2.373472146 | 0.9266914 | 0.0104302 | conserved hypothetical protein |
| C4B63_115g71 | -4.366167099 | 1.4114334 | 0.0019786 | retrotransposon hot spot protein (RHS) |
| C4B63_118g19 | -1.626882016 | 0.4722186 | 0.0005707 | retrotransposon hot spot (RHS) protein |
| C4B63_119g11 | -1.788589213 | 0.6827851 | 0.0088046 | unspecified product |
| C4B63_119g23 | -1.875157826 | 0.6259058 | 0.0027363 | retrotransposon hot spot protein (RHS) |
| C4B63_119g33 | -1.677519718 | 0.7074961 | 0.0177369 | retrotransposon hot spot protein (RHS) |
| C4B63_124g2 | -1.561239326 | 0.6254104 | 0.0125481 | phosphatidylinositol 3-kinase |
| C4B63_129g6 | -2.045892931 | 0.6141387 | 0.0008643 | retrotransposon hot spot (RHS) protein |
| C4B63_12g17 | -1.547570834 | 0.388778 | 6.874E-05 | dynein heavy chain |
| C4B63_12g175 | -1.554323941 | 0.1288758 | 1.705E-33 | Protein Associated with Differentiation |
| C4B63_12g196 | -1.632593897 | 0.5394578 | 0.0024752 | conserved hypothetical protein |
| C4B63_12g2035c | -1.878413558 | 0.4003685 | 2.709E-06 | zinc-finger protein 2 |
| C4B63_12g228 | -2.295289856 | 0.4584605 | 5.542E-07 | protein transport protein SEC61 subunit alpha |
| C4B63_12g230 | -1.887846078 | 0.464246 | 4.773E-05 | palmitoyl acyltransferase 7 |
| C4B63_12g330 | -1.738126556 | 0.4850401 | 0.0003391 | ATP synthase subunit beta, mitochondrial |
| C4B63_12g405 | -1.814984277 | 0.5558135 | 0.0010929 | cytochrome P450 |
| C4B63_130g21 | -2.099313473 | 1.0533397 | 0.0462607 | unspecified product |
| C4B63_133g41 | -1.991448352 | 0.5952483 | 0.0008211 | dynein heavy chain |
| C4B63_133g42 | -1.652253267 | 0.2387244 | 4.479E-12 | dynein heavy chain |
| C4B63_133g61 | -2.677939672 | 0.7303653 | 0.0002458 | heat shock protein 70 (hsp70) |
| C4B63_138g36 | -1.955141722 | 0.8403584 | 0.0199889 | conserved hypothetical protein |
| C4B63_13g113 | -1.52475179 | 0.2934145 | 2.03E-07 | zinc-finger of a C2HC-type |
| C4B63_13g238 | -1.519003756 | 0.3831919 | 7.368E-05 | dynein heavy chain |
| C4B63_13g296 | -2.009215915 | 0.6592987 | 0.0023075 | conserved hypothetical protein |
| C4B63_13g334 | -1.52216643 | 0.2102845 | 4.533E-13 | conserved hypothetical protein |
| C4B63_13g360 | -1.830378831 | 0.6687092 | 0.0061968 | unspecified product |
| C4B63_141g14 | -1.504809773 | 0.5162491 | 0.0035581 | retrotransposon hot spot (RHS) protein |
| C4B63_145g29 | -2.1032386 | 0.2018826 | 2.049E-25 | ABC transporter |
| C4B63_145g33 | -1.606750296 | 0.6608131 | 0.0150375 | permease-like protein |
| C4B63_147g45 | -1.692234115 | 0.6030468 | 0.0050139 | kynureninase |
| C4B63_149g41 | -1.59158905 | 0.7504655 | 0.0339384 | conserved hypothetical protein |
| C4B63_14g101 | -1.905614327 | 0.420301 | 5.79E-06 | conserved hypothetical protein |
| C4B63_14g16 | -1.671781206 | 0.3569502 | 2.82E-06 | DnaJ chaperone protein |
| C4B63_14g17 | -2.399544206 | 0.681827 | 0.0004327 | small G-protein |
| C4B63_14g246 | -2.204971184 | 0.4947666 | 8.327E-06 | microtubule-associated protein |
| C4B63_14g56 | -1.714316101 | 0.2281941 | 5.799E-14 | Flagellum attachment zone protein 5 |
| C4B63_151g41 | -2.125593492 | 0.9352995 | 0.0230482 | retrotransposon hot spot (RHS) protein |
| C4B63_151g43 | -1.696507044 | 0.5455743 | 0.0018735 | unspecified product |
| C4B63_152g25 | -1.663142654 | 0.8434279 | 0.0486228 | protein kinase |
| C4B63_153g40 | -1.948604977 | 0.5657167 | 0.0005721 | inositol 5-phosphatase 1 |
| C4B63_154g7 | -1.926564015 | 0.9813525 | 0.0496262 | retrotransposon hot spot protein (RHS) |
| C4B63_155g3 | -2.791360625 | 0.9379873 | 0.0029213 | retrotransposon hot spot protein (RHS) |
| C4B63_159g13 | -2.219989859 | 0.9274755 | 0.0166847 | retrotransposon hot spot protein (RHS) |
| C4B63_15g11 | -1.717634003 | 0.4070978 | 2.452E-05 | KRI1-like family/KRI1-like family C-terminal |
| C4B63_15g223 | -1.625709028 | 0.7994348 | 0.0419947 | unspecified product |
| C4B63_15g33c | -2.008089285 | 0.345085 | 5.916E-09 | SELENOT |
| C4B63_160g32 | -1.754690619 | 0.3862277 | 5.542E-06 | kinesin-C |
| C4B63_166g23 | -1.732792599 | 0.2785537 | 4.95E-10 | retrotransposon hot spot (RHS) protein |
| C4B63_16g102 | -1.671335715 | 0.3033418 | 3.594E-08 | conserved hypothetical protein |
| C4B63_16g11 | -2.652345697 | 0.4707214 | 1.754E-08 | 5'-AMP-activated protein kinase subunit beta |
| C4B63_16g121 | -2.248276861 | 0.5292226 | 2.154E-05 | dynein heavy chain |
| C4B63_16g136 | -3.456036922 | 0.6646049 | 1.991E-07 | conserved hypothetical protein |
| C4B63_16g157 | -2.009885398 | 0.3189689 | 2.954E-10 | conserved hypothetical protein |
| C4B63_16g194 | -1.995789417 | 0.7515536 | 0.0079179 | mannosyl-oligosaccharide 1,2-alpha-mannosidase IB |
| C4B63_16g293 | -1.651943661 | 0.432774 | 0.000135 | inositol 1,4,5-trisphosphate receptor |
| C4B63_16g295 | -1.514066289 | 0.3688307 | 4.042E-05 | RNA-binding protein |
| C4B63_16g331 | -1.78286189 | 0.2553895 | 2.932E-12 | ubiquitin-activating enzyme E1 |
| C4B63_16g34 | -1.552534755 | 0.6250834 | 0.0130017 | retrotransposon hot spot protein (RHS) |
| C4B63_16g6 | -1.529468308 | 0.1933059 | 2.53E-15 | permease-like protein |
| C4B63_16g691c | -4.926742143 | 1.2840305 | 0.0001246 | conserved hypothetical protein |
| C4B63_16g79 | -1.860053403 | 0.3967658 | 2.758E-06 | ammonium transporter |
| C4B63_16g94 | -1.573184683 | 0.2550247 | 6.883E-10 | hook complex protein, conserved |
| C4B63_16g95 | -1.651955799 | 0.5917996 | 0.0052479 | conserved hypothetical protein |
| C4B63_173g11 | -1.7187822 | 0.3482819 | 8.014E-07 | unspecified product |
| C4B63_173g8 | -2.18783542 | 0.8157826 | 0.0073209 | small GTP-binding protein RAB6 |
| C4B63_175g20 | -1.573046706 | 0.4876822 | 0.0012573 | dynein heavy chain |
| C4B63_17g226 | -2.067201697 | 0.416204 | 6.807E-07 | conserved hypothetical protein |
| C4B63_18g204 | -1.880992615 | 0.3355582 | 2.076E-08 | 3-hydroxy-3-methylglutaryl-CoA reductase |
| C4B63_18g221 | -2.143847315 | 0.4556281 | 2.535E-06 | protein kinase-like protein |
| C4B63_190nc21 | -2.403454398 | 0.7167895 | 0.0007992 | Large subunit ribosomal RNA,note=Large subunit ribosomal RNA, LSU-beta, (old nomenclature, 24S-beta) |
| C4B63_190nc23 | -1.794300206 | 0.7878318 | 0.0227554 | Large subunit ribosomal RNA,note=Large subunit ribosomal RNA, LSU-alpha, (old nomenclature, 24S-alpha) |
| C4B63_190nc25 | -2.12685743 | 0.8166831 | 0.0092072 | Large subunit ribosomal RNA,note=Large subunit ribosomal RNA, LSU-alpha, (old nomenclature, 24S-alpha) |
| C4B63_190nc28 | -5.940127762 | 1.2328331 | 1.448E-06 | Large subunit ribosomal RNA,note=Large subunit ribosomal RNA, LSU-beta, (old nomenclature, 24S-beta) |
| C4B63_190nc3 | -3.282469828 | 1.0298674 | 0.0014362 | 18S ribosomal RNA,note=SSU |
| C4B63_190nc30 | -2.191207773 | 0.9061459 | 0.0155991 | Large subunit ribosomal RNA,note=Large subunit ribosomal RNA, LSU-alpha, (old nomenclature, 24S-alpha) |
| C4B63_190nc35 | -3.983039075 | 0.847033 | 2.572E-06 | Large subunit ribosomal RNA,note=Large subunit ribosomal RNA, LSU-alpha, (old nomenclature, 24S-alpha) |
| C4B63_190nc6 | -2.494503986 | 1.0486054 | 0.0173654 | 18S ribosomal RNA,note=SSU |
| C4B63_191g13 | -1.778548284 | 0.6237247 | 0.0043514 | retrotransposon hot spot (RHS) protein |
| C4B63_191g34 | -1.560190956 | 0.7059475 | 0.0271006 | 3-methylcrotonyl-CoA carboxylase |
| C4B63_194g17 | -3.13381562 | 0.4312145 | 3.664E-13 | phosphate-repressible phosphate permease |
| C4B63_198g23 | -3.275760961 | 0.3141523 | 1.861E-25 | retrotransposon hot spot (RHS) protein |
| C4B63_198g24 | -1.583775479 | 0.4692148 | 0.0007372 | conserved hypothetical protein |
| C4B63_199g2 | -1.501781753 | 0.2714848 | 3.171E-08 | conserved hypothetical protein |
| C4B63_19g101 | -1.697429326 | 0.5079843 | 0.0008333 | U-rich RNA-binding protein UBP-2 |
| C4B63_19g103 | -1.637046967 | 0.4916476 | 0.0008693 | RNA-binding protein |
| C4B63_19g207 | -1.621764468 | 0.4154439 | 9.474E-05 | calpain-like cysteine peptidase |
| C4B63_19g217 | -2.000164734 | 0.5493795 | 0.0002718 | calpain cysteine peptidase |
| C4B63_19g218 | -1.687891528 | 0.2676363 | 2.851E-10 | calpain-like cysteine peptidase |
| C4B63_19g219 | -1.924125838 | 0.8106902 | 0.0176232 | calpain-like cysteine peptidase(fragment) |
| C4B63_19g220 | -1.887704137 | 0.5263474 | 0.0003352 | calpain-like cysteine peptidase |
| C4B63_19g221 | -1.861804596 | 0.5227714 | 0.0003689 | calpain-like cysteine peptidase(fragment) |
| C4B63_19g222 | -1.827839147 | 0.5247585 | 0.0004955 | calpain-like cysteine peptidase |
| C4B63_19g228 | -2.0343134 | 0.3647806 | 2.45E-08 | calpain-like cysteine peptidase |
| C4B63_19g230 | -1.677420174 | 0.4599685 | 0.0002655 | calpain-like cysteine peptidase |
| C4B63_19g266 | -2.249459586 | 0.4880664 | 4.048E-06 | conserved hypothetical protein |
| C4B63_19g267 | -1.764603112 | 0.3965944 | 8.611E-06 | Right handed beta helix region containing protein |
| C4B63_1g76 | -1.507209841 | 0.6621381 | 0.0228294 | MSP (Major sperm protein) domain containing protein |
| C4B63_1g896 | -1.623608923 | 0.362846 | 7.654E-06 | Mucin-associated surface protein (MASP) |
| C4B63_201g21 | -1.9288286 | 0.2477618 | 6.971E-15 | retrotransposon hot spot (RHS) protein |
| C4B63_207g22 | -2.316247223 | 0.7373605 | 0.0016822 | ATP-dependent RNA helicase HEL67 |
| C4B63_209g6 | -2.485929613 | 1.1628994 | 0.0325411 | retrotransposon hot spot (RHS) protein |
| C4B63_20g130 | -2.093709596 | 1.0534519 | 0.0468698 | unspecified product |
| C4B63_20g137 | -1.637542981 | 0.1794638 | 7.197E-20 | leucine-rich repeat protein (LRRP) |
| C4B63_20g16 | -1.722746679 | 0.5242457 | 0.0010157 | conserved hypothetical protein |
| C4B63_20g168 | -1.509228736 | 0.4192219 | 0.0003181 | mitogen activated protein kinase |
| C4B63_20g302 | -1.50354535 | 0.6801937 | 0.0270728 | Flagellar attachment zone protein 1 |
| C4B63_210g33 | -1.947376733 | 0.7594293 | 0.0103395 | p-nitrophenylphosphatase |
| C4B63_212g9 | -1.56975304 | 0.3330735 | 2.442E-06 | Protein Associated with Differentiation |
| C4B63_21g246 | -2.087694234 | 0.5002718 | 3.005E-05 | conserved hypothetical protein |
| C4B63_21g247 | -1.672215185 | 0.2669427 | 3.744E-10 | conserved hypothetical protein |
| C4B63_21g282 | -1.709442329 | 0.3311711 | 2.446E-07 | mitogen-activated protein kinase 3 |
| C4B63_21g47 | -1.954086076 | 0.7072731 | 0.00573 | Eukaryotic translation initiation factor 4 gamma 5 |
| C4B63_21g87 | -1.796944224 | 0.4211978 | 1.988E-05 | conserved hypothetical protein |
| C4B63_21g92 | -1.610774154 | 0.2179717 | 1.47E-13 | conserved hypothetical protein |
| C4B63_221g10 | -2.497830729 | 0.6761091 | 0.0002204 | ammonium transporter |
| C4B63_222g26 | -1.918160865 | 0.8843732 | 0.0300865 | UDP-Gal or UDP-GlcNAc-dependent glycosyltransferase |
| C4B63_22g113 | -1.733781448 | 0.4509179 | 0.0001206 | phosphatidylinositol 3-kinase |
| C4B63_22g138 | -2.075336559 | 0.1823561 | 5.219E-30 | unspecified product |
| C4B63_22g160 | -1.731750143 | 0.4840077 | 0.0003463 | unspecified product |
| C4B63_22g77 | -2.215985416 | 0.6144228 | 0.0003102 | conserved hypothetical protein |
| C4B63_22g78 | -1.533647627 | 0.2977089 | 2.584E-07 | protein kinase |
| C4B63_22nc10 | -1.96373998 | 0.6948497 | 0.0047113 | Large subunit ribosomal RNA, LSU-srRNA1,note=Old nomenclature 24S-S1 |
| C4B63_22nc11 | -1.642695714 | 0.1449124 | 8.725E-30 | Large subunit ribosomal RNA,note=Large subunit ribosomal RNA, LSU-beta, (old nomenclature, 24S-beta) |
| C4B63_22nc12 | -3.630777514 | 1.307272 | 0.0054801 | Large subunit ribosomal RNA, LSU-srRNA2,note=Old nomenclature 24S-S2 |
| C4B63_22nc3 | -2.071156013 | 0.7018729 | 0.0031685 | 18S ribosomal RNA,note=SSU |
| C4B63_22nc9 | -2.426571591 | 0.7904732 | 0.0021422 | Large subunit ribosomal RNA,note=Large subunit ribosomal RNA, LSU-alpha, (old nomenclature, 24S-alpha) |
| C4B63_234g10 | -1.70812414 | 0.3283548 | 1.971E-07 | Radial spoke head protein 9 homolog |
| C4B63_234g11 | -1.546288358 | 0.4305313 | 0.0003287 | conserved hypothetical protein |
| C4B63_238g9 | -1.946705759 | 0.5479374 | 0.0003812 | conserved hypothetical protein |
| C4B63_23g109 | -2.08533337 | 0.5052079 | 3.665E-05 | UDP-glucose:glycoprotein glucosyltransferase |
| C4B63_23g122 | -2.109480982 | 0.6821506 | 0.0019855 | serine/threonine protein kinase |
| C4B63_23g16 | -1.647997714 | 0.1756226 | 6.367E-21 | Battenin |
| C4B63_23g26 | -1.561116376 | 0.1928635 | 5.754E-16 | conserved hypothetical protein |
| C4B63_23g27 | -1.523575138 | 0.2794526 | 4.981E-08 | kinetoplastid-specific dual specificity phosphatase |
| C4B63_23g275 | -1.589213537 | 0.1780757 | 4.482E-19 | ATP-dependent DEAD/H RNA helicase |
| C4B63_23g28 | -1.628505251 | 0.1911057 | 1.575E-17 | kinetoplastid-specific dual specificity phosphatase |
| C4B63_246g15 | -1.838551161 | 0.7782496 | 0.0181561 | conserved hypothetical protein |
| C4B63_24g190 | -1.50461848 | 0.2015943 | 8.419E-14 | Exportin-7 |
| C4B63_24g194 | -2.289585092 | 0.8367973 | 0.0062167 | microtubule-associated protein |
| C4B63_24g196 | -2.193398549 | 0.4507148 | 1.136E-06 | microtubule-associated protein |
| C4B63_24g233 | -1.954740289 | 0.7370014 | 0.0079948 | conserved hypothetical protein |
| C4B63_24g236 | -1.814859298 | 0.7937078 | 0.0222216 | microtubule-associated protein Gb4 |
| C4B63_24g275 | -1.937014478 | 0.3611864 | 8.189E-08 | conserved hypothetical protein |
| C4B63_24g276 | -1.671771087 | 0.3051975 | 4.31E-08 | conserved hypothetical protein |
| C4B63_24g356 | -1.694486154 | 0.5318123 | 0.0014413 | retrotransposon hot spot (RHS) protein |
| C4B63_250g24 | -1.524640957 | 0.3201642 | 1.916E-06 | retrotransposon hot spot protein (RHS) |
| C4B63_250g25 | -1.851523378 | 0.3341518 | 3.008E-08 | retrotransposon hot spot (RHS) protein |
| C4B63_259g9 | -4.100294298 | 0.7747571 | 1.207E-07 | Ran-binding protein |
| C4B63_25g196 | -1.536329595 | 0.2584547 | 2.777E-09 | conserved hypothetical protein |
| C4B63_25g199 | -2.727320045 | 0.7563017 | 0.0003108 | unspecified product |
| C4B63_25g217 | -1.72820034 | 0.5097694 | 0.0006985 | membrane transporter protein |
| C4B63_25g237 | -1.911074695 | 0.517081 | 0.0002191 | eukaryotic translation initiation factor 3 subunit b |
| C4B63_25g256 | -2.101340046 | 0.2877782 | 2.836E-13 | conserved hypothetical protein |
| C4B63_25g321 | -1.500489146 | 0.3606386 | 3.173E-05 | conserved hypothetical protein |
| C4B63_267g21 | -1.791778721 | 0.3537833 | 4.092E-07 | conserved hypothetical protein |
| C4B63_267g6 | -1.971498295 | 0.5433138 | 0.0002849 | protein transport protein Sec24C(fragment) |
| C4B63_26g142 | -2.377637282 | 0.8601802 | 0.0057077 | retrotransposon hot spot (RHS) protein |
| C4B63_26g266 | -2.269537462 | 0.5832862 | 9.985E-05 | E1-like ubiquitin-activating enzyme |
| C4B63_26g305 | -1.736020638 | 0.5567254 | 0.0018192 | conserved hypothetical protein |
| C4B63_271g10 | -1.650282751 | 0.5661361 | 0.003557 | conserved hypothetical protein |
| C4B63_275g12 | -1.691578573 | 0.8407764 | 0.0442279 | Glucose-dependent insulinotropic receptor |
| C4B63_275g14 | -1.68178021 | 0.525366 | 0.0013688 | conserved hypothetical protein |
| C4B63_278g3 | -1.751478722 | 0.4873037 | 0.0003254 | conserved hypothetical protein |
| C4B63_279g16 | -2.782773863 | 0.9194787 | 0.0024743 | L1Tc protein fragment |
| C4B63_27g202 | -1.957189078 | 0.4542041 | 1.64E-05 | conserved hypothetical protein |
| C4B63_27g214 | -1.646784622 | 0.4401477 | 0.000183 | serine/threonine protein phosphatase |
| C4B63_27g219 | -1.713645811 | 0.2611096 | 5.276E-11 | Flagellum attachment zone protein 3 |
| C4B63_27g220 | -1.576550721 | 0.2456824 | 1.39E-10 | Flagellum attachment zone protein 3 |
| C4B63_282g15 | -2.241090418 | 0.4318709 | 2.111E-07 | cation transporter |
| C4B63_287g6 | -2.487644252 | 0.4392481 | 1.484E-08 | SLACS reverse transcriptase |
| C4B63_28g334 | -1.789451502 | 0.830092 | 0.031105 | expression site-associated gene (ESAG-like) protein |
| C4B63_292g78c | -1.550333179 | 0.2206445 | 2.12E-12 | calmodulin |
| C4B63_295g15 | -1.978801981 | 0.63617 | 0.0018678 | nucleosome assembly protein-like protein |
| C4B63_29g137 | -2.018763758 | 0.950184 | 0.0336198 | syntaxin binding protein |
| C4B63_29g220 | -2.593852489 | 0.7111146 | 0.0002647 | retrotransposon hot spot protein (RHS) |
| C4B63_29g4 | -2.232537739 | 0.4887887 | 4.936E-06 | double-strand-break repair protein rad21 homolog |
| C4B63_2g174 | -1.764333645 | 0.4667184 | 0.0001566 | polyadenylate-binding protein 1 |
| C4B63_2g292 | -2.241132661 | 0.6794918 | 0.0009729 | glyceraldehyde-3-phosphate dehydrogenase |
| C4B63_2g304 | -1.689554251 | 0.3381369 | 5.833E-07 | Protein FAM184A |
| C4B63_2g321 | -1.916939935 | 0.730187 | 0.008658 | conserved hypothetical protein |
| C4B63_2g33 | -1.595472325 | 0.2771374 | 8.564E-09 | conserved hypothetical protein |
| C4B63_2g433 | -1.88135864 | 0.6193805 | 0.0023856 | conserved hypothetical protein |
| C4B63_2g447 | -1.691797054 | 0.2875574 | 4.021E-09 | Unc104-like kinesin(fragment) |
| C4B63_2g450 | -1.650579632 | 0.3619471 | 5.109E-06 | metallo-peptidase, Clan MF, Family M17 |
| C4B63_2g455 | -1.56775172 | 0.4108242 | 0.0001356 | Cytoplasmic dynein 2 heavy chain (DYNC2H1) |
| C4B63_2g50 | -2.494135966 | 0.5341174 | 3.017E-06 | RNA-binding protein |
| C4B63_2g816 | -2.070962509 | 0.2582095 | 1.053E-15 | conserved hypothetical protein |
| C4B63_2g817 | -2.831160126 | 0.4003839 | 1.537E-12 | conserved hypothetical protein |
| C4B63_2g824 | -1.727899533 | 0.3825621 | 6.283E-06 | syntaxin |
| C4B63_303g16 | -1.576476825 | 0.3940127 | 6.305E-05 | trypanothione reductase |
| C4B63_306g13 | -1.598263784 | 0.6433381 | 0.0129796 | HPP family |
| C4B63_307g9 | -1.925155316 | 0.727557 | 0.0081437 | SLACS reverse transcriptase |
| C4B63_30g198 | -1.947195906 | 0.3654679 | 9.933E-08 | lanosterol synthase |
| C4B63_30g199 | -2.188271877 | 0.4019532 | 5.207E-08 | lanosterol synthase |
| C4B63_30g297 | -1.767614786 | 0.4105728 | 1.668E-05 | retrotransposon hot spot (RHS) protein |
| C4B63_310g2 | -1.868870659 | 0.3267131 | 1.064E-08 | L-2-hydroxyglutarate dehydrogenase, mitochondrial |
| C4B63_319g3 | -2.062634276 | 0.646963 | 0.0014317 | Casein kinase II subunit beta |
| C4B63_319g6 | -1.693409675 | 0.2749214 | 7.292E-10 | retrotransposon hot spot protein (RHS) |
| C4B63_31g238 | -1.739795709 | 0.8140898 | 0.0325894 | trans-sialidase, Group V |
| C4B63_31g80 | -1.527862467 | 0.3479149 | 1.126E-05 | Calcium ATPase SERCA-like |
| C4B63_323g5 | -1.776623739 | 0.7259786 | 0.0143966 | unspecified product |
| C4B63_32g185 | -2.415831734 | 1.1118805 | 0.0297996 | unspecified product |
| C4B63_32g219 | -1.546608525 | 0.6813441 | 0.0232114 | Trypanosome basal body component protein |
| C4B63_32g259 | -1.560118957 | 0.5711125 | 0.0063005 | Cyclin-U4-1 |
| C4B63_32g370 | -2.233987708 | 0.8488879 | 0.0084968 | unspecified product |
| C4B63_32g45 | -1.614002159 | 0.4139807 | 9.67E-05 | conserved hypothetical protein |
| C4B63_335nc1 | -2.232612917 | 0.9686573 | 0.0211748 | 18S ribosomal RNA,note=SSU |
| C4B63_335nc6 | -1.868163417 | 0.6582252 | 0.0045371 | Large subunit ribosomal RNA,note=Large subunit ribosomal RNA, LSU-alpha, (old nomenclature, 24S-alpha) |
| C4B63_335nc7 | -4.846814986 | 1.1552709 | 2.724E-05 | Large subunit ribosomal RNA, LSU-srRNA1,note=Old nomenclature 24S-S1 |
| C4B63_335nc8 | -1.612605094 | 0.6491416 | 0.0129839 | Large subunit ribosomal RNA,note=Large subunit ribosomal RNA, LSU-beta, (old nomenclature, 24S-beta) |
| C4B63_34g285 | -1.917404504 | 0.6153363 | 0.0018331 | imidazolonepropionase |
| C4B63_34g302 | -1.766648889 | 0.3294664 | 8.224E-08 | conserved hypothetical protein |
| C4B63_34g364 | -2.18860697 | 0.2710833 | 6.828E-16 | zinc-finger containing protein |
| C4B63_34g99 | -1.733717052 | 0.6050348 | 0.0041637 | unspecified product |
| C4B63_354g14 | -1.659563724 | 0.5137029 | 0.0012353 | retrotransposon hot spot (RHS) protein |
| C4B63_357g2 | -4.130924795 | 1.0276098 | 5.821E-05 | SLACS reverse transcriptase |
| C4B63_35g100 | -1.554822531 | 0.3762179 | 3.584E-05 | flagellar calcium-binding-like protein |
| C4B63_35g117 | -1.718882664 | 0.501404 | 0.0006077 | conserved hypothetical protein |
| C4B63_35g118 | -1.534031183 | 0.4390955 | 0.0004765 | conserved hypothetical protein |
| C4B63_35g119 | -1.844881991 | 0.2652975 | 3.55E-12 | Vacuolar protein sorting-associated protein 13C |
| C4B63_35g154 | -2.189733244 | 0.828285 | 0.0082006 | trans-sialidase, Group V |
| C4B63_365g5 | -2.197165387 | 0.6529889 | 0.000766 | SLACS reverse transcriptase (fragment) |
| C4B63_367g3 | -1.880498065 | 0.3336569 | 1.74E-08 | SLACS reverse transcriptase |
| C4B63_36g114 | -1.530137646 | 0.7066833 | 0.0303694 | Mucin-associated surface protein (MASP) |
| C4B63_370g11 | -2.420066894 | 0.6276464 | 0.0001154 | unspecified product |
| C4B63_389g6 | -2.816543362 | 0.3152251 | 4.071E-19 | SLACS reverse transcriptase |
| C4B63_389nc4 | -8.534415726 | 1.588107 | 7.703E-08 | spliced leader mini-exon transcript,ncRNA class=other |
| C4B63_38g160 | -2.289024935 | 0.6676597 | 0.0006071 | ADP-ribosylation factor |
| C4B63_38g171 | -1.512892746 | 0.5631623 | 0.0072221 | conserved hypothetical protein |
| C4B63_38g172 | -1.674772848 | 0.5399373 | 0.0019235 | conserved hypothetical protein |
| C4B63_38g173 | -1.998809302 | 0.5696868 | 0.0004505 | conserved hypothetical protein |
| C4B63_38g222 | -1.78032398 | 0.8638947 | 0.039321 | UDP-Gal or UDP-GlcNAc-dependent glycosyltransferase |
| C4B63_392g23 | -3.222450365 | 1.2668637 | 0.0109703 | retrotransposon hot spot (RHS) protein |
| C4B63_39g332 | -1.80973 | 0.7660406 | 0.0181548 | conserved hypothetical protein |
| C4B63_39g344 | -1.525095604 | 0.50813 | 0.0026875 | 14-3-3 protein 1 |
| C4B63_39g367 | -1.637171337 | 0.6611023 | 0.0132705 | retrotransposon hot spot protein (RHS) |
| C4B63_3g4692c | -1.500011965 | 0.2983118 | 4.948E-07 | ubiquitin-conjugating enzyme E2 |
| C4B63_3g833 | -1.692586047 | 0.7714598 | 0.0282351 | Mucin-associated surface protein (MASP) |
| C4B63_406g4 | -3.274482103 | 1.1190609 | 0.0034324 | kinesin-like protein(fragment) |
| C4B63_406g6 | -1.674337623 | 0.4193473 | 6.532E-05 | kinesin-like protein |
| C4B63_40g107 | -1.871658453 | 0.5673233 | 0.0009699 | conserved hypothetical protein |
| C4B63_40g112 | -2.136830817 | 0.5305549 | 5.636E-05 | conserved hypothetical protein |
| C4B63_40g123 | -1.986070689 | 0.5359086 | 0.0002106 | Pre-mRNA-splicing factor CWC22 homolog |
| C4B63_40g124 | -1.755017138 | 0.4775163 | 0.0002376 | Pre-mRNA-splicing factor CWC22 |
| C4B63_40g130 | -1.937502893 | 0.2945901 | 4.802E-11 | conserved hypothetical protein |
| C4B63_40g150 | -3.17110476 | 1.3528452 | 0.0190768 | conserved hypothetical protein |
| C4B63_40g160 | -2.323475262 | 0.2806038 | 1.229E-16 | conserved hypothetical protein |
| C4B63_40g190 | -1.563971847 | 0.5530029 | 0.0046819 | conserved hypothetical protein |
| C4B63_40g52 | -2.203372373 | 0.4704514 | 2.82E-06 | conserved hypothetical protein |
| C4B63_40g92 | -2.327710213 | 0.5615739 | 3.398E-05 | kinetoplastid kinetochore protein 2 |
| C4B63_40g97 | -1.548401921 | 0.2424365 | 1.694E-10 | eukaryotic translation initiation factor 4 gamma 4 |
| C4B63_41g205 | -1.626566919 | 0.2174432 | 7.408E-14 | alanine aminotransferase |
| C4B63_41g208 | -1.792887374 | 0.4026863 | 8.495E-06 | ser/thr protein phosphatase |
| C4B63_41g218 | -2.253274011 | 0.3665684 | 7.899E-10 | Protein NLRC3 |
| C4B63_41g248 | -1.690373549 | 0.6192018 | 0.0063349 | cyclin-like F-box protein |
| C4B63_42g180 | -1.624815379 | 0.1991686 | 3.406E-16 | nitrate reductase |
| C4B63_42g187 | -2.353145796 | 0.4832749 | 1.121E-06 | conserved hypothetical protein |
| C4B63_43g161 | -1.553562445 | 0.2337029 | 2.979E-11 | ATP-dependent RNA helicase SUB2 |
| C4B63_43g211 | -1.614653787 | 0.5504475 | 0.0033533 | conserved hypothetical protein |
| C4B63_43g213 | -1.611155827 | 0.4325454 | 0.0001955 | Golgin subfamily A member 5 |
| C4B63_43g5 | -1.744886225 | 0.5912538 | 0.0031658 | cdc2-related kinase 1 |
| C4B63_44g146 | -2.060121041 | 0.5991177 | 0.0005847 | Vacuolar proton pyrophosphatase 1 |
| C4B63_44g179 | -1.87646653 | 0.7064137 | 0.0078997 | RNA-binding protein |
| C4B63_44g183 | -1.568632366 | 0.2760607 | 1.33E-08 | conserved hypothetical protein |
| C4B63_44g195 | -2.436262855 | 0.7914221 | 0.0020816 | Coiled-coil domain-containing protein 39 |
| C4B63_44g211 | -1.828197879 | 0.154591 | 2.864E-32 | mevalonate kinase |
| C4B63_44g221 | -2.335132796 | 0.6042337 | 0.0001113 | cytoskeleton-associated protein CAP5.5 |
| C4B63_44g235 | -1.664534343 | 0.2489234 | 2.279E-11 | Kinesin-13 4 |
| C4B63_45g103 | -1.875008975 | 0.3097828 | 1.425E-09 | protein kinase |
| C4B63_45g123 | -1.50562951 | 0.3454388 | 1.309E-05 | chaperone protein DNAj |
| C4B63_45g132 | -1.534736772 | 0.5800026 | 0.0081429 | conserved hypothetical protein |
| C4B63_45g232 | -1.681163757 | 0.3761082 | 7.826E-06 | ubiquitin-protein ligase-like |
| C4B63_45g234 | -2.03685952 | 0.6891983 | 0.0031226 | ubiquitin-protein ligase-like |
| C4B63_45g235 | -2.012024562 | 0.5946981 | 0.0007163 | ubiquitin-protein ligase-like |
| C4B63_45g236 | -1.807598703 | 0.5543184 | 0.0011104 | ubiquitin-protein ligase |
| C4B63_45g238 | -1.872906082 | 0.2009883 | 1.18E-20 | glutamamyl carboxypeptidase |
| C4B63_46g189 | -1.501423424 | 0.4001002 | 0.000175 | retrotransposon hot spot (RHS) protein |
| C4B63_476nc1 | -3.751204631 | 0.6567628 | 1.119E-08 | 18S ribosomal RNA,note=SSU |
| C4B63_476nc14 | -2.060098496 | 0.8927933 | 0.0210283 | Large subunit ribosomal RNA, LSU-srRNA3,note=Old nomenclature 24S-S6 |
| C4B63_476nc7 | -2.334512019 | 0.500649 | 3.117E-06 | Large subunit ribosomal RNA,note=Large subunit ribosomal RNA, LSU-alpha, (old nomenclature, 24S-alpha) |
| C4B63_476nc8 | -1.840530581 | 0.8408998 | 0.028614 | Large subunit ribosomal RNA, LSU-srRNA1,note=Old nomenclature 24S-S1 |
| C4B63_47g163 | -2.235550905 | 1.0033493 | 0.0258746 | retrotransposon hot spot (RHS) protein |
| C4B63_47g165 | -1.593874123 | 0.5693255 | 0.0051169 | unspecified product |
| C4B63_47g24 | -1.649110554 | 0.4175947 | 7.846E-05 | Glucose-6-phosphate dehydrogenase |
| C4B63_47g71 | -1.782078491 | 0.4515506 | 7.928E-05 | Voltage-dependent calcium channel subunit |
| C4B63_47g73 | -1.945914155 | 0.5091026 | 0.0001322 | importin beta-1 subunit |
| C4B63_483g4 | -2.085970648 | 0.3004775 | 3.861E-12 | retrotransposon hot spot (RHS) protein |
| C4B63_48g122 | -1.666157483 | 0.5477534 | 0.0023517 | neurobeachin/beige protein |
| C4B63_48g132 | -1.897289177 | 0.2021181 | 6.171E-21 | universal minicircle sequence binding protein |
| C4B63_49g113 | -1.873768589 | 0.4168195 | 6.944E-06 | F-box and WD40 domain containing protein |
| C4B63_49g168 | -2.237329874 | 0.3384066 | 3.808E-11 | conserved hypothetical protein |
| C4B63_49g176 | -2.204265068 | 0.3882847 | 1.371E-08 | serine/threonine protein phosphatase |
| C4B63_49g64 | -1.635563303 | 0.3454594 | 2.196E-06 | Intraflagellar transport protein 74 homolog |
| C4B63_49g71 | -2.515157022 | 0.8009457 | 0.0016881 | Flagellar attachment zone protein 10 |
| C4B63_4g127 | -1.787509709 | 0.5574497 | 0.0013432 | U3 small nucleolar RNA-associated protein 14 homolog A |
| C4B63_4g188 | -1.667838158 | 0.5095769 | 0.0010642 | Elongation factor Tu, mitochondrial |
| C4B63_4g249 | -1.515495902 | 0.2141065 | 1.46E-12 | conserved hypothetical protein |
| C4B63_4g356 | -1.617747697 | 0.3219669 | 5.045E-07 | RNA-binding protein 29 |
| C4B63_4g506 | -1.585539308 | 0.4278661 | 0.0002108 | Microtubule Associated kinase (CAP-Gly domain) |
| C4B63_4g69 | -1.513200557 | 0.3492853 | 1.476E-05 | mitogen-activated protein kinase kinase kinase |
| C4B63_4g90 | -1.975132683 | 0.3905038 | 4.239E-07 | ATP-dependent RNA helicase HEL67 |
| C4B63_4g99 | -1.761928024 | 0.2434004 | 4.527E-13 | conserved hypothetical protein |
| C4B63_511nc3 | -1.950923182 | 0.3742038 | 1.853E-07 | 18S ribosomal RNA,note=SSU |
| C4B63_511nc6 | -1.565640693 | 0.2214854 | 1.563E-12 | Large subunit ribosomal RNA, LSU-srRNA2,note=Old nomenclature 24S-S2 |
| C4B63_511nc7 | -1.917130187 | 0.8119311 | 0.018216 | Large subunit ribosomal RNA,note=Large subunit ribosomal RNA, LSU-beta, (old nomenclature, 24S-beta) |
| C4B63_511nc8 | -2.402643509 | 1.191577 | 0.0437628 | Large subunit ribosomal RNA, LSU-srRNA1,note=Old nomenclature 24S-S1 |
| C4B63_511nc9 | -1.636758832 | 0.4479885 | 0.0002586 | Large subunit ribosomal RNA,note=Large subunit ribosomal RNA, LSU-alpha, (old nomenclature, 24S-alpha) |
| C4B63_51g112 | -1.829214118 | 0.4887138 | 0.0001819 | conserved hypothetical protein |
| C4B63_51g219 | -1.717896327 | 0.75638 | 0.0231344 | unspecified product |
| C4B63_522nc7 | -2.300834569 | 0.4328051 | 1.06E-07 | Large subunit ribosomal RNA,note=Large subunit ribosomal RNA, LSU-alpha, (old nomenclature, 24S-alpha) |
| C4B63_52g135 | -2.222904956 | 0.7711367 | 0.0039437 | kinesin-like protein |
| C4B63_52g147 | -1.809279767 | 0.2003969 | 1.741E-19 | conserved hypothetical protein |
| C4B63_52g170 | -2.246179246 | 0.7088327 | 0.0015305 | cytoskeleton associated protein |
| C4B63_53g146 | -1.684565025 | 0.423552 | 6.972E-05 | conserved hypothetical protein |
| C4B63_53g152 | -1.820530107 | 0.7298836 | 0.0126215 | conserved hypothetical protein |
| C4B63_54g24 | -1.765606393 | 0.5943512 | 0.0029717 | ATP-dependent DEAD/H RNA helicase |
| C4B63_54g35 | -1.502880737 | 0.5891154 | 0.010739 | protein kinase |
| C4B63_55g55 | -1.90905877 | 0.7687707 | 0.0130185 | Paraflagellar rod protein 2 |
| C4B63_56g160 | -1.841097773 | 0.6744992 | 0.0063416 | retrotransposon hot spot (RHS) protein |
| C4B63_577nc1 | -3.18595972 | 0.5259569 | 1.383E-09 | 18S ribosomal RNA,note=SSU |
| C4B63_581nc1 | -2.436478759 | 0.84098 | 0.0037652 | 18S ribosomal RNA,note=SSU |
| C4B63_58g126 | -2.160229987 | 0.3212739 | 1.769E-11 | ubiquitin-conjugating enzyme variant Kua homologue |
| C4B63_58g130 | -1.853401386 | 0.2675147 | 4.262E-12 | vacuolar protein sorting-associated protein 35 |
| C4B63_58g145 | -2.393509131 | 0.7126532 | 0.0007834 | R27-2 protein |
| C4B63_59g178 | -2.13192073 | 0.8541374 | 0.0125605 | unspecified product |
| C4B63_5g352 | -1.947621538 | 0.5108573 | 0.0001376 | Present in the outer mitochondrial membrane proteome 10 |
| C4B63_60g115 | -1.659952195 | 0.6645899 | 0.0124999 | unspecified product |
| C4B63_60g141 | -1.575268348 | 0.3088959 | 3.402E-07 | conserved hypothetical protein |
| C4B63_61g136 | -1.832892875 | 0.3630211 | 4.441E-07 | ER membrane protein complex subunit 2 |
| C4B63_621g1 | -2.597272193 | 0.6835445 | 0.0001449 | retrotransposon hot spot (RHS) protein |
| C4B63_621g2 | -2.552179104 | 0.6959315 | 0.0002451 | retrotransposon hot spot (RHS) protein |
| C4B63_65g17 | -1.892758894 | 0.5958797 | 0.0014911 | retrotransposon hot spot protein (RHS) |
| C4B63_66g5 | -1.600615522 | 0.575038 | 0.0053777 | retrotransposon hot spot (RHS) protein |
| C4B63_68g100 | -3.291505524 | 0.8962489 | 0.0002402 | unspecified product |
| C4B63_68g109 | -1.946144587 | 0.3364244 | 7.26E-09 | retrotransposon hot spot protein (RHS) |
| C4B63_68g123 | -1.932773926 | 0.8961415 | 0.0310233 | retrotransposon hot spot protein (RHS) |
| C4B63_68g81 | -2.054292634 | 0.4812018 | 1.963E-05 | retrotransposon hot spot protein (RHS) |
| C4B63_69g157 | -2.290885385 | 0.6714967 | 0.0006458 | retrotransposon hot spot (RHS) protein |
| C4B63_69g167 | -2.341556834 | 1.0673447 | 0.0282487 | retrotransposon hot spot (RHS) protein |
| C4B63_69g62 | -1.612528211 | 0.4399387 | 0.000247 | Endonuclease/Exonuclease/phosphatase family |
| C4B63_6g138 | -1.565986294 | 0.5666877 | 0.0057202 | conserved hypothetical protein |
| C4B63_6g214 | -2.577989565 | 0.4664083 | 3.252E-08 | 2,3-bisphosphoglycerate-independent phosphoglycerate mutase |
| C4B63_6g236 | -2.150855195 | 0.1971541 | 1.038E-27 | cation transporter |
| C4B63_6g258 | -1.674452992 | 0.2991081 | 2.166E-08 | Vacuolar proton pump subunit B |
| C4B63_6g309 | -3.11236723 | 0.9597296 | 0.0011829 | heat shock protein 70 (hsp70) |
| C4B63_6g514 | -1.825271615 | 0.2522959 | 4.667E-13 | carbonic anhydrase-like protein |
| C4B63_6g608 | -2.75769189 | 0.9614037 | 0.0041255 | retrotransposon hot spot (RHS) protein |
| C4B63_6g609 | -1.760207527 | 0.709843 | 0.0131488 | retrotransposon hot spot (RHS) protein |
| C4B63_70g95 | -1.874732455 | 0.5420766 | 0.0005433 | 14-3-3 protein 2 |
| C4B63_72g79 | -1.577039159 | 0.5378415 | 0.0033661 | inositol 5-phosphatase 1 |
| C4B63_73g67 | -3.823650816 | 1.5056229 | 0.0110985 | unspecified product |
| C4B63_74g4 | -1.647415824 | 0.6165339 | 0.0075387 | conserved hypothetical protein |
| C4B63_75g68 | -2.017183289 | 0.4875564 | 3.514E-05 | calcium motive p-type ATPase |
| C4B63_76g23 | -2.377853769 | 0.7057066 | 0.0007531 | cytoskeleton associated protein |
| C4B63_77g27 | -1.812384199 | 0.6097039 | 0.0029532 | conserved hypothetical protein |
| C4B63_77g75 | -2.150503747 | 0.3007801 | 8.693E-13 | ABC transporter |
| C4B63_78g11 | -1.739126086 | 0.3364919 | 2.361E-07 | conserved hypothetical protein |
| C4B63_78g25 | -3.020372861 | 0.2238504 | 1.724E-41 | nucleolar RNA-binding protein |
| C4B63_78g28 | -1.68690442 | 0.4371053 | 0.0001137 | RNA recognition motif (a.k.a. RRM, RBD, or RNP domain) |
| C4B63_78g9 | -1.875243507 | 0.4883089 | 0.0001229 | conserved hypothetical protein |
| C4B63_79g1 | -2.011681599 | 0.400679 | 5.149E-07 | R27-2 protein |
| C4B63_79g113 | -2.814079094 | 0.437478 | 1.255E-10 | Atrophin-1 family domain |
| C4B63_79g39 | -2.062283601 | 0.5528833 | 0.0001914 | conserved hypothetical protein |
| C4B63_80g47 | -1.643776943 | 0.3118433 | 1.356E-07 | amino acid transporter |
| C4B63_80g59 | -1.646097451 | 0.2554661 | 1.167E-10 | retrotransposon hot spot protein (RHS) |
| C4B63_82g70 | -1.674114461 | 0.1530568 | 7.597E-28 | dispersed gene family protein 1 (DGF-1) |
| C4B63_82g81 | -1.952065411 | 0.1738482 | 2.953E-29 | dispersed gene family protein 1 (DGF-1) |
| C4B63_82g85 | -1.962083813 | 0.1699893 | 8.065E-31 | dispersed gene family protein 1 (DGF-1) |
| C4B63_82nc5 | -1.519210223 | 0.6080903 | 0.012478 | C/D small nucleolar RNA (snoRNA),ncRNA class=snoRNA |
| C4B63_85g38 | -2.761653691 | 0.7158953 | 0.0001145 | retrotransposon hot spot (RHS) protein |
| C4B63_85g50 | -2.252893709 | 0.6259586 | 0.0003193 | conserved hypothetical protein |
| C4B63_85g55 | -1.540659863 | 0.2593435 | 2.84E-09 | fatty acyl CoA syntetase 1 |
| C4B63_85g66 | -1.608353337 | 0.2830912 | 1.336E-08 | Dynein 18 kDa light chain, flagellar outer arm |
| C4B63_85g83 | -1.946385255 | 0.4315158 | 6.465E-06 | Eukaryotic initiation factor 4A-1 |
| C4B63_86g56 | -3.527573729 | 1.0449217 | 0.0007357 | glyceraldehyde-3-phosphate dehydrogenase |
| C4B63_88g108 | -1.797099645 | 0.5660495 | 0.0014993 | trans-sialidase, Group V |
| C4B63_8g194 | -1.661946483 | 0.2446971 | 1.107E-11 | conserved hypothetical protein |
| C4B63_90g50 | -1.657152057 | 0.3355205 | 7.85E-07 | signal recognition particle(fragment) |
| C4B63_93g2 | -2.330706635 | 0.7779704 | 0.0027365 | retrotransposon hot spot protein (RHS) |
| C4B63_95g34 | -1.768420463 | 0.1780635 | 3.04E-23 | mitochondrial processing peptidase alpha subunit |
| C4B63_97g24 | -1.903282405 | 0.3084049 | 6.77E-10 | chaperone DNAJ protein |
| C4B63_97g34 | -4.657025919 | 1.6214924 | 0.004078 | P-type H+-ATPase |
| C4B63_9g235 | -1.975122026 | 0.6590134 | 0.0027257 | RNA-binding protein |
| C4B63_9g249 | -1.879145957 | 0.4006872 | 2.735E-06 | conserved hypothetical protein |
| C4B63_9g272 | -1.94035771 | 0.6260173 | 0.0019383 | NUP-1 protein |
| C4B63_9g305 | -1.778681211 | 0.2675539 | 2.972E-11 | Signal recognition particle receptor subunit beta |
| C4B63_9g319 | -1.761027915 | 0.688486 | 0.0105329 | heterogeneous nuclear ribonucleoprotein H/F |
| C4B63_9g322 | -1.738575894 | 0.4318334 | 5.673E-05 | translation initiation factor IF-2 |
| C4B63_9g327 | -1.725931537 | 0.2336537 | 1.505E-13 | conserved hypothetical protein |
| C4B63_9g328 | -1.944786906 | 0.264614 | 1.989E-13 | conserved hypothetical protein |
| C4B63_9g329 | -1.668518478 | 0.3809223 | 1.186E-05 | Beige/BEACH domain |
| C4B63_9g376 | -2.386033 | 0.6622953 | 0.000315 | Present in the outer mitochondrial membrane proteome 22 |
| C4B63_9g377 | -1.629120188 | 0.4626617 | 0.0004296 | Present in the outer mitochondrial membrane proteome 22 |
| C4B63_9g382 | -1.824029296 | 0.7258325 | 0.0119704 | conserved hypothetical protein |
| C4B63_9g415 | -1.608090381 | 0.5477991 | 0.0033296 | conserved hypothetical protein |
