# Supplementary material for: Transcriptomic and ultrastructural responses to Amiodarone–Itraconazole in naturally benznidazole-resistant and -susceptible Trypanosoma cruzi strains
Source: PLoS Negl Trop Dis. 2026 Jan 14;20(1):e0013916. doi: 10.1371/journal.pntd.0013916 (PMC12863684; doi:10.1371/journal.pntd.0013916)
Supplement: S3 Table — (DOCX) [file pntd.0013916.s004.docx]

**S3 Table.** Gene Ontology (GO) enrichment analysis for the DA strain, showing significantly enriched terms across the three GO categories: Biological Process (BP), Cellular Component (CC), and Molecular Function (MF).

| **GO ID** | **GO Term** | **Genes with this term** | **Fold enrichment** | **P-value** |
| --- | --- | --- | --- | --- |
| UP-REGULATED GENES (BP) | | | | |
| GO:0120029 | proton export across plasma membrane | 1 | 118.99 | 0.0083769 |
| GO:0140115 | export across plasma membrane | 1 | 118.99 | 0.0083769 |
| GO:0006888 | endoplasmic reticulum to Golgi vesicle-mediated transport | 1 | 66.93 | 0.0148498 |
| GO:0006810 | transport | 3 | 5.27 | 0.0153459 |
| GO:0051234 | establishment of localization | 3 | 5.25 | 0.0154835 |
| GO:0051179 | localization | 3 | 5.16 | 0.0162533 |
| GO:0055085 | transmembrane transport | 2 | 8.37 | 0.0221116 |
| GO:0009405 | obsolete pathogenesis | 3 | 4.52 | 0.0231341 |
| GO:0048193 | Golgi vesicle transport | 1 | 23.8 | 0.0412738 |
| GO:1902600 | proton transmembrane transport | 1 | 22.78 | 0.043073 |
| GO:0140352 | export from cell | 1 | 22.31 | 0.0439716 |
| GO:0098655 | cation transmembrane transport | 1 | 19.83 | 0.0493472 |
| GO:0098660 | inorganic ion transmembrane transport | 1 | 19.83 | 0.0493472 |
| GO:0098662 | inorganic cation transmembrane transport | 1 | 19.83 | 0.0493472 |
| UP-REGULATED GENES (CC) | | | | |
| GO:0016020 | membrane | 7 | 2.7 | 0.0021404 |
| GO:0030127 | COPII vesicle coat | 1 | 178.48 | 0.0055915 |
| GO:0030134 | COPII-coated ER to Golgi transport vesicle | 1 | 178.48 | 0.0055915 |
| GO:0012507 | ER to Golgi transport vesicle membrane | 1 | 178.48 | 0.0055915 |
| GO:0030133 | transport vesicle | 1 | 133.86 | 0.0074492 |
| GO:0030658 | transport vesicle membrane | 1 | 133.86 | 0.0074492 |
| GO:0031224 | intrinsic component of membrane | 6 | 2.58 | 0.0094476 |
| GO:0016021 | integral component of membrane | 6 | 2.58 | 0.0094476 |
| GO:0030120 | vesicle coat | 1 | 76.49 | 0.0130042 |
| GO:0030662 | coated vesicle membrane | 1 | 71.39 | 0.0139274 |
| GO:0030135 | coated vesicle | 1 | 66.93 | 0.0148498 |
| GO:0030659 | cytoplasmic vesicle membrane | 1 | 42.84 | 0.0231177 |
| GO:0012506 | vesicle membrane | 1 | 42.84 | 0.0231177 |
| GO:0030117 | membrane coat | 1 | 36.93 | 0.0267728 |
| GO:0048475 | coated membrane | 1 | 36.93 | 0.0267728 |
| GO:0110165 | cellular anatomical entity | 7 | 1.79 | 0.0308053 |
| GO:0005575 | cellular component | 7 | 1.76 | 0.0334166 |
| GO:0031410 | cytoplasmic vesicle | 1 | 28.18 | 0.034953 |
| GO:0097708 | intracellular vesicle | 1 | 28.18 | 0.034953 |
| GO:0031982 | vesicle | 1 | 26.77 | 0.0367627 |
| UP-REGULATED GENES (MF) | | | | |
| GO:0008553 | P-type proton-exporting transporter activity | 1 | 118.99 | 0.0083769 |
| GO:0015662 | P-type ion transporter activity | 1 | 97.35 | 0.0102301 |
| GO:0140358 | P-type transmembrane transporter activity | 1 | 97.35 | 0.0102301 |
| GO:0009678 | pyrophosphate hydrolysis-driven proton transmembrane transporter activity | 1 | 66.93 | 0.0148498 |
| GO:0019829 | ATPase-coupled cation transmembrane transporter activity | 1 | 50.99 | 0.0194506 |
| GO:0004308 | exo-alpha-sialidase activity | 3 | 4.52 | 0.0232215 |
| GO:0016997 | alpha-sialidase activity | 3 | 4.52 | 0.0232215 |
| GO:0022857 | transmembrane transporter activity | 2 | 7.76 | 0.025468 |
| GO:0004553 | hydrolase activity, hydrolyzing O-glycosyl compounds | 3 | 4.34 | 0.0259321 |
| GO:0016798 | hydrolase activity, acting on glycosyl bonds | 3 | 4.29 | 0.0265895 |
| GO:0005215 | transporter activity | 2 | 7.26 | 0.0288429 |
| GO:0022853 | active ion transmembrane transporter activity | 1 | 30.6 | 0.032233 |
| GO:0015078 | proton transmembrane transporter activity | 1 | 21 | 0.0466627 |
| DOWN-REGULATED GENES (BP) | | | | |
| GO:0006486 | protein glycosylation | 3 | 10.55 | 0.0026929 |
| GO:0009100 | glycoprotein metabolic process | 3 | 10.55 | 0.0026929 |
| GO:0009101 | glycoprotein biosynthetic process | 3 | 10.55 | 0.0026929 |
| GO:0043413 | macromolecule glycosylation | 3 | 10.55 | 0.0026929 |
| GO:0070085 | glycosylation | 3 | 10.55 | 0.0026929 |
| GO:0006464 | cellular protein modification process | 5 | 3.23 | 0.0162337 |
| GO:0036211 | protein modification process | 5 | 3.23 | 0.0162337 |
| GO:1901137 | carbohydrate derivative biosynthetic process | 3 | 5.42 | 0.0169652 |
| GO:0043412 | macromolecule modification | 5 | 2.92 | 0.0241597 |
| GO:0019538 | protein metabolic process | 7 | 2.22 | 0.028791 |
| GO:1901135 | carbohydrate derivative metabolic process | 3 | 4.28 | 0.0313803 |
| GO:0044267 | cellular protein metabolic process | 6 | 2.35 | 0.0347607 |
| GO:0006888 | endoplasmic reticulum to Golgi vesicle-mediated transport | 1 | 25.5 | 0.0385405 |
| GO:0006749 | glutathione metabolic process | 1 | 24 | 0.0409017 |
| DOWN-REGULATED GENES (CC) | | | | |
| GO:0012505 | endomembrane system | 5 | 7.53 | 0.0004096 |
| GO:0005794 | Golgi apparatus | 4 | 9.83 | 0.0006289 |
| GO:0000139 | Golgi membrane | 3 | 9.27 | 0.0038816 |
| GO:0098588 | bounding membrane of organelle | 3 | 6.72 | 0.009464 |
| GO:0005788 | endoplasmic reticulum lumen | 1 | 101.99 | 0.0097708 |
| GO:0030008 | TRAPP complex | 1 | 58.28 | 0.0170391 |
| GO:0005783 | endoplasmic reticulum | 2 | 9.6 | 0.0180794 |
| GO:0043227 | membrane-bounded organelle | 6 | 2.46 | 0.0282768 |
| GO:0031090 | organelle membrane | 3 | 4.31 | 0.0308208 |
| GO:0044444 | obsolete cytoplasmic part | 2 | 7.09 | 0.0317687 |
| GO:0005737 | cytoplasm | 5 | 2.51 | 0.0428712 |
| GO:0043231 | intracellular membrane-bounded organelle | 5 | 2.43 | 0.0485522 |
| DOWN-REGULATED GENES (MF) | | | | |
| GO:0016740 | transferase activity | 9 | 2.87 | 0.001945 |
| GO:0003964 | RNA-directed DNA polymerase activity | 3 | 10.37 | 0.0028269 |
| GO:0034061 | DNA polymerase activity | 3 | 8.93 | 0.0043092 |
| GO:0016757 | glycosyltransferase activity | 3 | 7.7 | 0.006527 |
| GO:0005525 | GTP binding | 3 | 7.16 | 0.007978 |
| GO:0019001 | guanyl nucleotide binding | 3 | 7.12 | 0.0081069 |
| GO:0032561 | guanyl ribonucleotide binding | 3 | 7.12 | 0.0081069 |
| GO:0140097 | catalytic activity, acting on DNA | 3 | 6.44 | 0.0106405 |
| GO:0016779 | nucleotidyltransferase activity | 3 | 5.61 | 0.0154116 |
| GO:0003674 | molecular function | 20 | 1.27 | 0.0178064 |
| GO:0016772 | transferase activity, transferring phosphorus-containing groups | 5 | 2.87 | 0.0256686 |
| GO:0003824 | catalytic activity | 14 | 1.45 | 0.0472112 |
