# Supplementary material for: Transcriptomic and ultrastructural responses to Amiodarone–Itraconazole in naturally benznidazole-resistant and -susceptible Trypanosoma cruzi strains
Source: PLoS Negl Trop Dis. 2026 Jan 14;20(1):e0013916. doi: 10.1371/journal.pntd.0013916 (PMC12863684; doi:10.1371/journal.pntd.0013916)
Supplement: S4 Table — (DOCX) [file pntd.0013916.s005.docx]

**S4 Table.** Gene Ontology (GO) enrichment analysis for the MG strain, showing significantly enriched terms across the three GO categories: Biological Process (BP), Cellular Component (CC), and Molecular Function (MF).

| **GO ID** | **GO Term** | **Genes with this term** | **Fold enrichment** | **P-value** |
| --- | --- | --- | --- | --- |
| UP-REGULATED GENES (BP) | | | | |
| GO:0009405 | obsolete pathogenesis | 9 | 5.72 | 7.581E-06 |
| GO:0007155 | cell adhesion | 3 | 14.09 | 0.0011601 |
| GO:0022610 | biological adhesion | 3 | 14.09 | 0.0011601 |
| GO:0006486 | protein glycosylation | 2 | 7.77 | 0.0267481 |
| GO:0009100 | glycoprotein metabolic process | 2 | 7.77 | 0.0267481 |
| GO:0009101 | glycoprotein biosynthetic process | 2 | 7.77 | 0.0267481 |
| GO:0043413 | macromolecule glycosylation | 2 | 7.77 | 0.0267481 |
| GO:0070085 | glycosylation | 2 | 7.77 | 0.0267481 |
| GO:0006508 | proteolysis | 3 | 4.25 | 0.0315882 |
| UP-REGULATED GENES (CC) | | | | |
| GO:0016020 | membrane | 15 | 2.44 | 4.09E-05 |
| GO:0016021 | integral component of membrane | 13 | 2.36 | 0.0004269 |
| GO:0031224 | intrinsic component of membrane | 13 | 2.36 | 0.0004269 |
| GO:0005886 | plasma membrane | 3 | 10.57 | 0.0026501 |
| GO:0071944 | cell periphery | 3 | 9.8 | 0.0032804 |
| GO:0110165 | cellular anatomical entity | 15 | 1.61 | 0.0074837 |
| GO:0005575 | cellular component | 15 | 1.59 | 0.0087108 |
| GO:0000139 | Golgi membrane | 2 | 6.83 | 0.0339528 |
| UP-REGULATED GENES (MF) | | | | |
| GO:0004308 | exo-alpha-sialidase activity | 9 | 5.71 | 7.669E-06 |
| GO:0016997 | alpha-sialidase activity | 9 | 5.71 | 7.669E-06 |
| GO:0004553 | hydrolase activity, hydrolyzing O-glycosyl compounds | 9 | 5.48 | 1.078E-05 |
| GO:0016798 | hydrolase activity, acting on glycosyl bonds | 9 | 5.43 | 1.164E-05 |
| GO:0016787 | hydrolase activity | 12 | 2.91 | 0.0001142 |
| GO:0004222 | metalloendopeptidase activity | 3 | 11.66 | 0.002001 |
| GO:0008237 | metallopeptidase activity | 3 | 8.96 | 0.004228 |
| GO:0004175 | endopeptidase activity | 3 | 6.66 | 0.0096003 |
| GO:0003824 | catalytic activity | 14 | 1.6 | 0.0140563 |
| GO:0016757 | glycosyltransferase activity | 2 | 5.67 | 0.0476154 |
| DOWN-REGULATED GENES (BP) | | | | |
| GO:0007018 | microtubule-based movement | 17 | 5.56 | 8.148E-09 |
| GO:0006928 | movement of cell or subcellular component | 17 | 5.38 | 1.336E-08 |
| GO:0007017 | microtubule-based process | 17 | 4.12 | 7.066E-07 |
| GO:0006811 | ion transport | 13 | 3.87 | 2.906E-05 |
| GO:0072488 | ammonium transmembrane transport | 2 | 40.22 | 0.0006153 |
| GO:0015696 | ammonium transport | 2 | 40.22 | 0.0006153 |
| GO:0006812 | cation transport | 9 | 3.58 | 0.0008838 |
| GO:0006810 | transport | 28 | 1.85 | 0.0011324 |
| GO:0051234 | establishment of localization | 28 | 1.84 | 0.0011901 |
| GO:0051179 | localization | 28 | 1.81 | 0.0015557 |
| GO:0003341 | cilium movement | 3 | 12.07 | 0.0015981 |
| GO:0098660 | inorganic ion transmembrane transport | 6 | 4.47 | 0.0021016 |
| GO:0098662 | inorganic cation transmembrane transport | 6 | 4.47 | 0.0021016 |
| GO:0098655 | cation transmembrane transport | 6 | 4.47 | 0.0021016 |
| GO:0120031 | plasma membrane bounded cell projection assembly | 4 | 6.44 | 0.0031177 |
| GO:0030031 | cell projection assembly | 4 | 6.44 | 0.0031177 |
| GO:0060271 | cilium assembly | 4 | 6.44 | 0.0031177 |
| GO:0055085 | transmembrane transport | 14 | 2.2 | 0.0046577 |
| GO:0034220 | ion transmembrane transport | 6 | 3.77 | 0.0049697 |
| GO:0044782 | cilium organization | 4 | 5.55 | 0.0054172 |
| GO:0120036 | plasma membrane bounded cell projection organization | 4 | 5.55 | 0.0054172 |
| GO:0006816 | calcium ion transport | 2 | 16.09 | 0.0058557 |
| GO:0070925 | organelle assembly | 4 | 4.47 | 0.011739 |
| GO:0030001 | metal ion transport | 4 | 4.47 | 0.011739 |
| GO:0070286 | axonemal dynein complex assembly | 2 | 11.49 | 0.0118991 |
| GO:0035082 | axoneme assembly | 2 | 8.94 | 0.0197413 |
| GO:0001578 | microtubule bundle formation | 2 | 8.94 | 0.0197413 |
| GO:0030030 | cell projection organization | 4 | 3.66 | 0.0232104 |
| GO:0001932 | regulation of protein phosphorylation | 2 | 7.31 | 0.0291925 |
| GO:0008299 | isoprenoid biosynthetic process | 2 | 7.31 | 0.0291925 |
| GO:0045859 | regulation of protein kinase activity | 2 | 7.31 | 0.0291925 |
| GO:0033875 | ribonucleoside bisphosphate metabolic process | 2 | 6.7 | 0.0344662 |
| GO:0033865 | nucleoside bisphosphate metabolic process | 2 | 6.7 | 0.0344662 |
| GO:0034032 | purine nucleoside bisphosphate metabolic process | 2 | 6.7 | 0.0344662 |
| GO:0042325 | regulation of phosphorylation | 2 | 6.7 | 0.0344662 |
| GO:0043648 | dicarboxylic acid metabolic process | 2 | 6.7 | 0.0344662 |
| GO:0043549 | regulation of kinase activity | 2 | 6.7 | 0.0344662 |
| GO:0071705 | nitrogen compound transport | 10 | 1.84 | 0.0453163 |
| GO:0016567 | protein ubiquitination | 2 | 5.75 | 0.0460063 |
| GO:0009310 | amine catabolic process | 1 | 20.11 | 0.0491104 |
| GO:0006586 | indolalkylamine metabolic process | 1 | 20.11 | 0.0491104 |
| GO:0070189 | kynurenine metabolic process | 1 | 20.11 | 0.0491104 |
| GO:0097052 | L-kynurenine metabolic process | 1 | 20.11 | 0.0491104 |
| GO:0097053 | L-kynurenine catabolic process | 1 | 20.11 | 0.0491104 |
| GO:0006569 | tryptophan catabolic process | 1 | 20.11 | 0.0491104 |
| GO:0006568 | tryptophan metabolic process | 1 | 20.11 | 0.0491104 |
| GO:0007623 | circadian rhythm | 1 | 20.11 | 0.0491104 |
| GO:0007030 | Golgi organization | 1 | 20.11 | 0.0491104 |
| GO:0046218 | indolalkylamine catabolic process | 1 | 20.11 | 0.0491104 |
| GO:0048511 | rhythmic process | 1 | 20.11 | 0.0491104 |
| GO:0046874 | quinolinate metabolic process | 1 | 20.11 | 0.0491104 |
| GO:0043650 | dicarboxylic acid biosynthetic process | 1 | 20.11 | 0.0491104 |
| GO:0043420 | anthranilate metabolic process | 1 | 20.11 | 0.0491104 |
| GO:0042537 | benzene-containing compound metabolic process | 1 | 20.11 | 0.0491104 |
| GO:0042436 | indole-containing compound catabolic process | 1 | 20.11 | 0.0491104 |
| GO:0042430 | indole-containing compound metabolic process | 1 | 20.11 | 0.0491104 |
| GO:0042402 | cellular biogenic amine catabolic process | 1 | 20.11 | 0.0491104 |
| GO:0042182 | ketone catabolic process | 1 | 20.11 | 0.0491104 |
| GO:0032922 | circadian regulation of gene expression | 1 | 20.11 | 0.0491104 |
| GO:0034627 | 'de novo' NAD biosynthetic process | 1 | 20.11 | 0.0491104 |
| GO:0034354 | 'de novo' NAD biosynthetic process from tryptophan | 1 | 20.11 | 0.0491104 |
| GO:0019805 | quinolinate biosynthetic process | 1 | 20.11 | 0.0491104 |
| GO:0036159 | inner dynein arm assembly | 1 | 20.11 | 0.0491104 |
| DOWN-REGULATED GENES (CC) | | | | |
| GO:0005875 | microtubule associated complex | 7 | 8.04 | 1.979E-05 |
| GO:0030286 | dynein complex | 7 | 8.04 | 1.979E-05 |
| GO:0005874 | microtubule | 11 | 4.61 | 2.397E-05 |
| GO:0099512 | supramolecular fiber | 11 | 4.61 | 2.397E-05 |
| GO:0099081 | supramolecular polymer | 11 | 4.61 | 2.397E-05 |
| GO:0099080 | supramolecular complex | 11 | 4.61 | 2.397E-05 |
| GO:0099513 | polymeric cytoskeletal fiber | 11 | 4.61 | 2.397E-05 |
| GO:0015630 | microtubule cytoskeleton | 12 | 4.06 | 3.744E-05 |
| GO:0005856 | cytoskeleton | 13 | 3.11 | 0.0002736 |
| GO:0098796 | membrane protein complex | 6 | 2.74 | 0.0220494 |
| GO:0005744 | TIM23 mitochondrial import inner membrane translocase complex | 1 | 40.22 | 0.0248628 |
| GO:0030906 | retromer, cargo-selective complex | 1 | 40.22 | 0.0248628 |
| GO:1902494 | catalytic complex | 8 | 2.27 | 0.0251156 |
| GO:0044424 | obsolete intracellular part | 13 | 1.83 | 0.0254169 |
| GO:0031201 | SNARE complex | 1 | 20.11 | 0.0491104 |
| GO:0030904 | retromer complex | 1 | 20.11 | 0.0491104 |
| GO:0008278 | cohesin complex | 1 | 20.11 | 0.0491104 |
| GO:0005956 | protein kinase CK2 complex | 1 | 20.11 | 0.0491104 |
| GO:0031588 | nucleotide-activated protein kinase complex | 1 | 20.11 | 0.0491104 |
| DOWN-REGULATED GENES (MF) | | | | |
| GO:0004198 | calcium-dependent cysteine-type endopeptidase activity | 7 | 13.41 | 4.602E-07 |
| GO:0003777 | microtubule motor activity | 13 | 5.18 | 1.141E-06 |
| GO:0140657 | ATP-dependent activity | 27 | 2.71 | 1.946E-06 |
| GO:0008569 | minus-end-directed microtubule motor activity | 7 | 10.43 | 3.095E-06 |
| GO:0003774 | cytoskeletal motor activity | 13 | 4.12 | 1.505E-05 |
| GO:0035639 | purine ribonucleoside triphosphate binding | 55 | 1.73 | 1.98E-05 |
| GO:0032555 | purine ribonucleotide binding | 55 | 1.72 | 2.373E-05 |
| GO:0017076 | purine nucleotide binding | 55 | 1.71 | 2.483E-05 |
| GO:0036094 | small molecule binding | 60 | 1.65 | 2.995E-05 |
| GO:0032553 | ribonucleotide binding | 55 | 1.69 | 4.032E-05 |
| GO:0000166 | nucleotide binding | 58 | 1.65 | 4.1E-05 |
| GO:1901265 | nucleoside phosphate binding | 58 | 1.65 | 4.1E-05 |
| GO:0097367 | carbohydrate derivative binding | 55 | 1.68 | 4.589E-05 |
| GO:0043168 | anion binding | 57 | 1.65 | 4.94E-05 |
| GO:0005524 | ATP binding | 48 | 1.74 | 6.716E-05 |
| GO:0032559 | adenyl ribonucleotide binding | 48 | 1.73 | 8.047E-05 |
| GO:0030554 | adenyl nucleotide binding | 48 | 1.73 | 8.229E-05 |
| GO:0022890 | inorganic cation transmembrane transporter activity | 10 | 4.1 | 0.0001518 |
| GO:0015318 | inorganic molecular entity transmembrane transporter activity | 11 | 3.66 | 0.0002016 |
| GO:0008324 | cation transmembrane transporter activity | 10 | 3.66 | 0.0003923 |
| GO:0097159 | organic cyclic compound binding | 78 | 1.39 | 0.0005488 |
| GO:1901363 | heterocyclic compound binding | 78 | 1.39 | 0.0005488 |
| GO:0008519 | ammonium transmembrane transporter activity | 2 | 40.22 | 0.0006153 |
| GO:0009987 | cellular process | 2 | 40.22 | 0.0006153 |
| GO:0043167 | ion binding | 73 | 1.41 | 0.0006259 |
| GO:0022804 | active transmembrane transporter activity | 9 | 3.62 | 0.0008225 |
| GO:0004197 | cysteine-type endopeptidase activity | 7 | 4.4 | 0.0009932 |
| GO:0022857 | transmembrane transporter activity | 16 | 2.33 | 0.0014007 |
| GO:0015075 | ion transmembrane transporter activity | 10 | 3.05 | 0.0016263 |
| GO:0140033 | acetylation-dependent protein binding | 2 | 26.81 | 0.0018157 |
| GO:0070628 | proteasome binding | 2 | 26.81 | 0.0018157 |
| GO:0070577 | lysine-acetylated histone binding | 2 | 26.81 | 0.0018157 |
| GO:0042393 | histone binding | 2 | 26.81 | 0.0018157 |
| GO:0016504 | peptidase activator activity | 2 | 26.81 | 0.0018157 |
| GO:0005488 | binding | 115 | 1.23 | 0.0020278 |
| GO:0015399 | primary active transmembrane transporter activity | 7 | 3.8 | 0.0023344 |
| GO:0005215 | transporter activity | 16 | 2.18 | 0.0027597 |
| GO:0015631 | tubulin binding | 8 | 3.25 | 0.0031542 |
| GO:0042626 | ATPase-coupled transmembrane transporter activity | 6 | 3.77 | 0.0049697 |
| GO:0140030 | modification-dependent protein binding | 2 | 16.09 | 0.0058557 |
| GO:0061134 | peptidase regulator activity | 2 | 16.09 | 0.0058557 |
| GO:0008017 | microtubule binding | 7 | 3.2 | 0.0061532 |
| GO:0009678 | pyrophosphate hydrolysis-driven proton transmembrane transporter activity | 3 | 7.54 | 0.0066804 |
| GO:0022853 | active ion transmembrane transporter activity | 4 | 4.6 | 0.0106372 |
| GO:0046873 | metal ion transmembrane transporter activity | 4 | 4.6 | 0.0106372 |
| GO:0008092 | cytoskeletal protein binding | 8 | 2.64 | 0.0109421 |
| GO:0015085 | calcium ion transmembrane transporter activity | 2 | 11.49 | 0.0118991 |
| GO:0019829 | ATPase-coupled cation transmembrane transporter activity | 3 | 5.75 | 0.0144839 |
| GO:0003743 | translation initiation factor activity | 5 | 3.41 | 0.0152947 |
| GO:0008234 | cysteine-type peptidase activity | 7 | 2.66 | 0.0162775 |
| GO:0004496 | mevalonate kinase activity | 1 | 40.22 | 0.0248628 |
| GO:0004420 | hydroxymethylglutaryl-CoA reductase (NADPH) activity | 1 | 40.22 | 0.0248628 |
| GO:0004089 | carbonate dehydratase activity | 1 | 40.22 | 0.0248628 |
| GO:0046537 | 2,3-bisphosphoglycerate-independent phosphoglycerate mutase activity | 1 | 40.22 | 0.0248628 |
| GO:0050480 | imidazolonepropionase activity | 1 | 40.22 | 0.0248628 |
| GO:0140358 | P-type transmembrane transporter activity | 2 | 7.31 | 0.0291925 |
| GO:0015662 | P-type ion transporter activity | 2 | 7.31 | 0.0291925 |
| GO:0005261 | cation channel activity | 3 | 4.16 | 0.034436 |
| GO:0020037 | heme binding | 2 | 6.7 | 0.0344662 |
| GO:0008641 | ubiquitin-like modifier activating enzyme activity | 2 | 6.7 | 0.0344662 |
| GO:0046906 | tetrapyrrole binding | 2 | 6.7 | 0.0344662 |
| GO:0005515 | protein binding | 40 | 1.32 | 0.0362441 |
| GO:0030234 | enzyme regulator activity | 4 | 3.09 | 0.0397578 |
| GO:0005388 | P-type calcium transporter activity | 1 | 20.11 | 0.0491104 |
| GO:0046527 | glucosyltransferase activity | 1 | 20.11 | 0.0491104 |
| GO:0004127 | cytidylate kinase activity | 1 | 20.11 | 0.0491104 |
| GO:0003980 | UDP-glucose:glycoprotein glucosyltransferase activity | 1 | 20.11 | 0.0491104 |
| GO:0016822 | hydrolase activity, acting on acid carbon-carbon bonds | 1 | 20.11 | 0.0491104 |
| GO:0030429 | kynureninase activity | 1 | 20.11 | 0.0491104 |
| GO:0035251 | UDP-glucosyltransferase activity | 1 | 20.11 | 0.0491104 |
| GO:0004619 | phosphoglycerate mutase activity | 1 | 20.11 | 0.0491104 |
| GO:0016823 | hydrolase activity, acting on acid carbon-carbon bonds, in ketonic substances | 1 | 20.11 | 0.0491104 |
